# Distinctive Collembola communities in the Mesovoid Shallow Substratum: First data for the Sierra de Guadarrama National Park (Central Spain) and a description of two new species of *Orchesella* (Entomobryidae)

**DOI:** 10.1371/journal.pone.0189205

**Published:** 2017-12-13

**Authors:** Enrique Baquero, Enrique Ledesma, José D. Gilgado, Vicente M. Ortuño, Rafael Jordana

**Affiliations:** 1 Departamento de Biología Animal, Universidad de Navarra, Pamplona, Spain; 2 Departamento de Ciencias de la Vida, Facultad de Biología Ciencias Ambientales y Química, Universidad de Alcalá, Alcalá de Henares, Madrid, Spain; CHINA

## Abstract

Two new species of the genus *Orchesella* Templeton, 1836 have been identified following intensive sampling in the Colluvial *Milieu Souterrain Superficiel* (Mesovoid Shallow Substratum, or MSS) of the Sierra de Guadarrama using Subterranean Sampling Devices (SSD). The data were obtained from the first extraction of the traps between May and October of 2015. During a study of the Collembola taxon, 32 different genera (61 species) were identified. The highest representative genus presence in almost all traps was *Orchesella*, with two new species. One of the two species described had been misidentified until this study was carried out, indicating that their preferential habitat had not been sampled; the second species had never been identified. The community of the *Orchesella* species in the Colluvial MSS was investigated, leading to the conclusion that this environment has its own assemblage of characteristic species. The opportunity to study specimens that belong to five species of the genus *Orchesella*, including three previously recollected, has allowed for obtaining reliable information regarding their macrochaetotaxy. A part of this chaetotaxy is proposed as a useful diagnostic tool for the species of the genus. In conclusion, it can be affirmed that this study has demonstrated that the Colluvial Mesovoid Shallow Substratum (Colluvial MSS) has its own fauna, and it supports the hypothesis that it constitutes a new biotope, at least for Collembola.

## Introduction

The study of subterranean habitats has generally focused on caves, pits and even mines (of anthropogenic origin) [1, 2] although shallow subterranean environments [3] and specifically the *Milieu Souterrain Superficiel* (MSS), have become relevant in recent years [4]. This habitat was discovered in the 1980s and was described as suitable for hypogean species [5, 6, 7, 8]. The MSS is constituted by the fissure networks and interstices of rocky debris, usually under a soil layer, whose genesis is the product of different lithological processes in various types of rocks, i.e. bedrock, colluvial, volcanic or alluvial MSS [9, 5, 6, 10, 11, 12]. Thus, the MSS is a widely extended subterranean environment, although it has not been investigated sufficiently.

Some of the conditions of the MSS are similar to those usually present in caves (absence of light, high humidity and small variations of temperature) [13, 14] however, its close relation with the surface and the layers of the soil allows for wider temperature oscillations [4] and facilitates the entry of organic matter into the system [15]. The MSS can harbor fauna coming from the surface when external conditions are momentarily adverse [16, 17], but it also contains stable resident fauna. This resident fauna includes stenoic and hygrophilous as well as truly hypogean (or troglobiont sensu Sket [18]) species, i.e. species that are highly specialized and adapted to the subterranean environment and unable to complete their lifecycles outside of it [11, 19, 20, 21, 13, 12]

The most notable studies on the MSS have been carried out in Europe, including in the Iberian Peninsula and the Canary Islands, by several groups of researchers [4]. Although comprehensive lists of the Collembola species in the MSS are scarce, some studies have provided extensive lists of Collembola species [11]; however, few have recorded the presence of the *Orchesella* species in this environment. This genus was recorded in the MSS in Eastern Europe as *Orchesella carpatica* Ionesco, 1915 and *O*. *pontica* Ionesco, 1915 [22]; *O*. *carpatica*, *O*. *pontica* and *O*. *alticola* (Uzel, 1891) [16]; and *O*. *pontica* [17]. In addition, this genus has been recorded twice in the MSS of the Iberian Peninsula as *Orchesella bifasciata* Nicolet, 1842 from an alluvial MSS in eastern Spain[12], and undetermined species of *Orchesella* in a Colluvial MSS from that region [14].

In recent years, there has been a series of interesting findings of fauna in the MSS in several mountainous massifs of the Iberian Peninsula [23, 24, 25]. Recent samplings have revealed new species of *Orchesella* in the Colluvial MSS of Sierra de Guadarrama (Central Spain), which are described in this paper.

The Holarctic genus of collembola *Orchesella* Templeton, 1835 (Entomobryidae) includes 61 species that are among the largest collembolans [26]. These species inhabit the forest litter and are well-known for their key ecological role of recycling decaying organic matter [27]. Despite their size, the morphological characters of taxonomic importance are reduced mainly due to the disposition of sensorial hairs (chaetotaxy) and to pigmentation patterns [28, 29]. In addition, high levels of intraspecific variability affect both chaetotaxy and pigmentation and are sometimes correlated with their lifecycles [30, 31]. The use of these characteristics in combination with the methodologies used by other authors [32, 33] have proven to be useful in the identification of species that belong to the family of Entomobryidae.

## Material and methods

### Area of study

**Fig 1:**
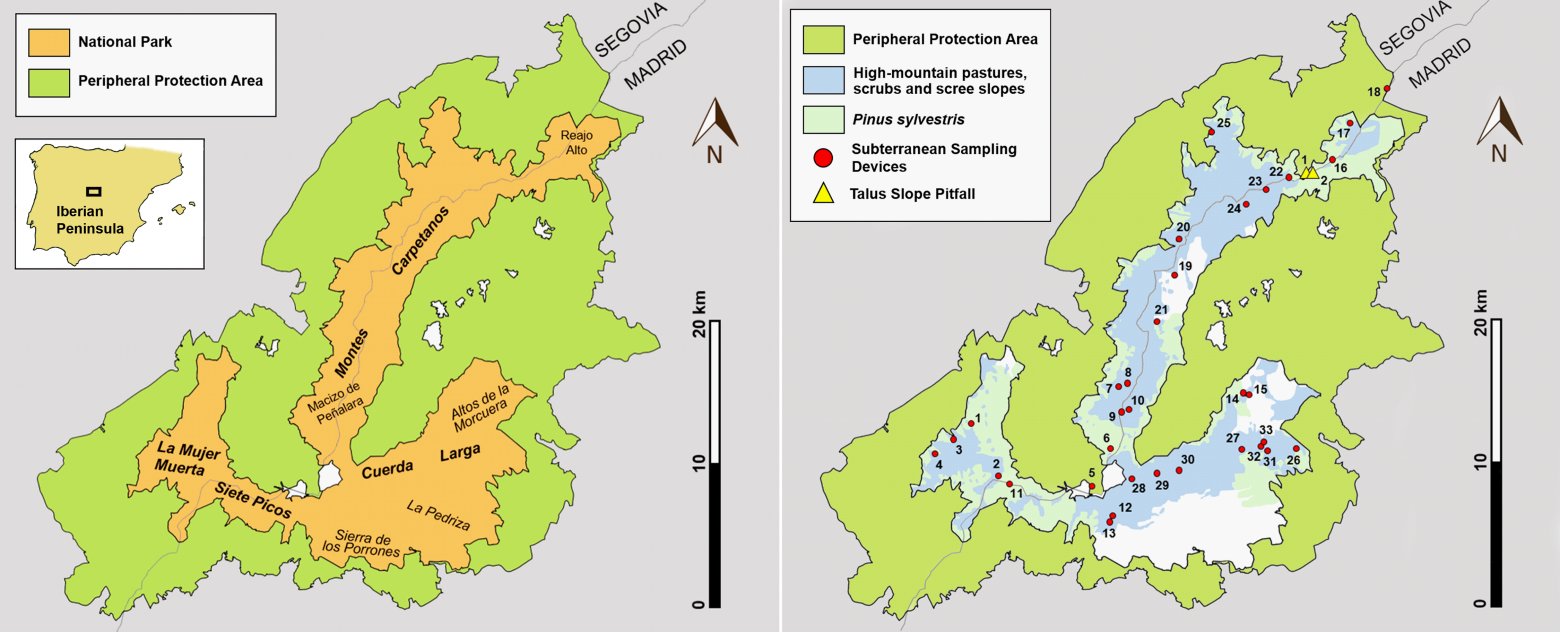
Maps of the Sierra de Guadarrama National Park. (A) basic orography. (B) habitats that occupy greater surfaces [36]. The spatial location of the sampling points is indicated and numbered.

**Fig 2:**
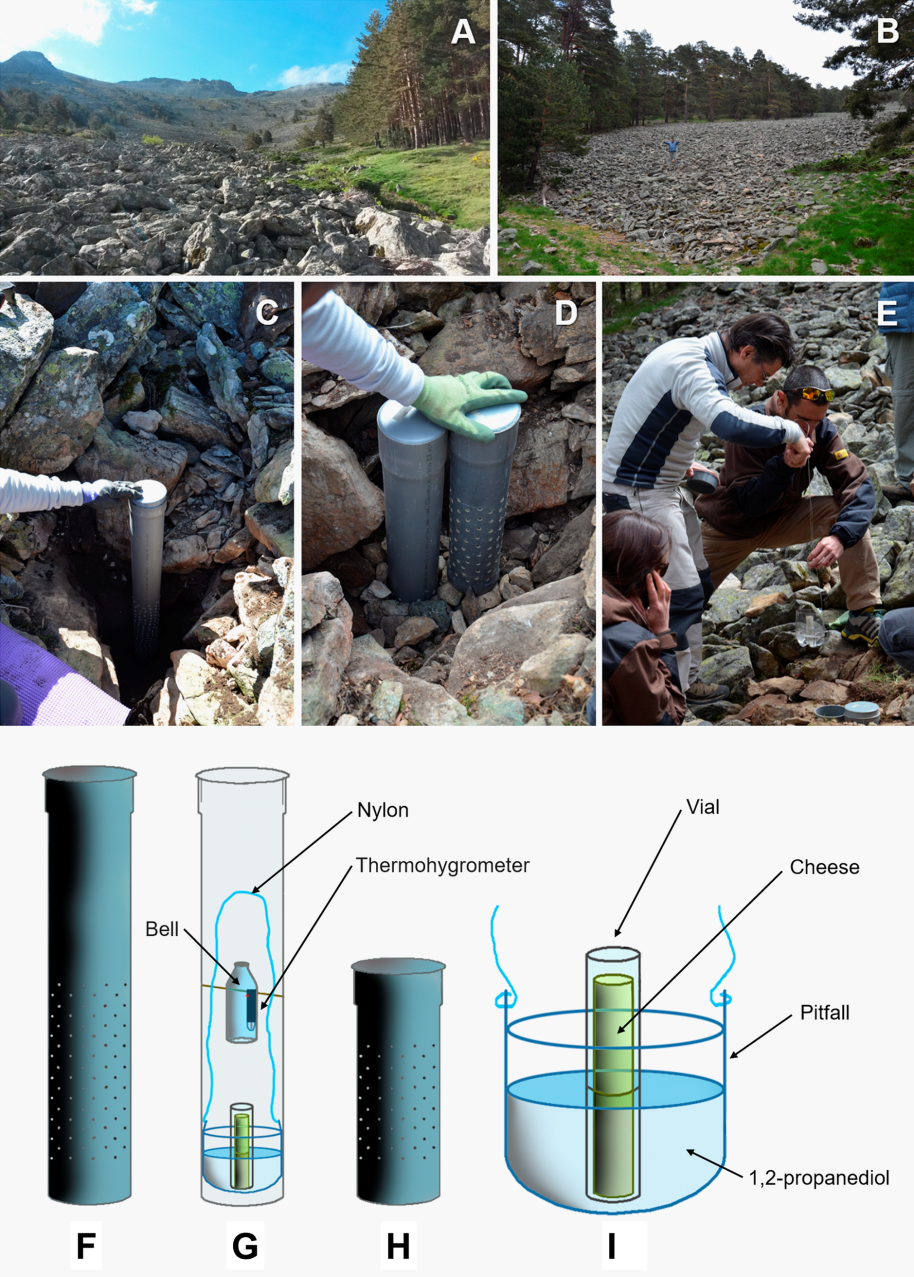
Installation of Subterranean Sampling Device (SSD). (A) Scree slopes from shadowed areas of La Mujer Muerta (Segovia). (B) Scree slopes from Cancho del Río Peces (Segovia). (C) SSD before being buried. (D) SSDs of 1 m (left) and of 0.5 m (right) in the process of installation. (E) Placing of the pitfall trap inside the cylinder (F–I) SSD used in sampling.

The study was conducted in the eastern half of the Central System (Iberian Peninsula) in the mountainous subunit called Sierra de Guadarrama, and more specifically within the limits of the protection area (33,960 hectares). A few years ago, it was declared a national park [34], and it has a belt of 62,687 hectares of Peripheral Protection Area [35] (Fig 1). The lower altitudinal limit is around 1200–1300 m a. s. l. (above sea level) on the slopes near the Puerto de la Morcuera, and the highest limit is located at the 2428 m altitude of the summit of Peñalara [36]. The park extends over three mountain ranges (Fig 1) with numerous peaks that exceed 2000 m a. s. l. Each of these ranges converge in a sector characterised by the presence of two natural passages, the Puerto de Los Cotos and the Puerto de Navacerrada. Both have ski slopes because these areas are outside the limits of the park and ZPP. The cord known as Montes Carpetanos (Fig 1) runs in an N–NE direction and serves as a natural borderline between the provinces of Segovia and Madrid. Its most southern end reaches the Puerto de Los Cotos, which constitutes the foothills of the emblematic Macizo de Peñalara, a maximum expression of the glacier model in the Sierra de Guadarrama. It should be noted that the NE boundary of the park only reaches the vicinity of the Reajo Alto peak. A second range, which includes Siete Picos and La Mujer Muerta (Fig 1), runs from the Puerto de Navacerrada in the NW–N direction, entering the province of Segovia. Finally, a third range that runs by the entire province of Madrid consists of a more complex configuration because it is formed by a set of mountains that are not arranged in line but are grouped in an almost parallel formation (Fig 1). The primary axis is Cuerda Larga, which begins in the vicinity of Puerto de Navacerrada, progressing along 16 km in an E–NE orientation until it reaches the Puerto de la Morcuera. To the south, lying on the mountainous buttress, are the mountains Sierra de los Porrones and Sierra de la Pedriza, and the fields of the Altos de la Morcuera are towards the north. The Sierra de Guadarrama is formed by rocks of different origins, but the lithological substrate of the national park is mostly comprised of gneiss metamorphic rocks [37]. This type of primitive rock has an igneous origin, and thus it is more specifically an orthogneis [38–37]. This geological material is the oldest of this orography [39], although there is controversy regarding its isotopic age which is said to be between the lower Ordovician and the middle Cambrian [40]. This type of rock is present in the three mountainous ranges and shows a certain tendency to fracture, and it is the main protagonist of the morrenic and colluvial deposits that originated from glacial events [41] and periglaciares [42], forming most of the scree-talus of the Sierra de Guadarrama. The granite, plutonic rock, which is associated with filonian rocks, also forms part of these reliefs; however, within the national park, it is limited to only a part of Siete Picos and almost all of La Pedriza. The massive character of these materials and their erosion due to disintegration does not favour the formation of scree-talus, and therefore these substrates have been excluded from underground surveys (Fig 2).

The park consists of three bioclimatic floors, which respond to the typical model of a mountainous Mediterranean enclave: the supramediterranean, oromediterranean and crioromediterranean floors. On the supramediterranean floor (1300–1700 m a. s. l.), the melojo oak (*Quercus pyrenaica* Willd.) is the most common species, although it has been depleted in favour of the Scots’ pine (*Pinus sylvestris* L.), which mostly grows in the upper supramediterranean floor but is also found in the oromediterranean and the crioromediterranean floors of some south-facing slopes [43]. These pine forests, whether natural or naturalised, comprise the second most extensive vegetation unit of the park, forming dense forest masses with little fragmentation [36] (Fig 2). The oromediterranean floor (1700–2200 m a. s. l.) is characterised by the presence of *Juniperus communis alpina* (Suter) Celak. (creeping juniper) and shrubs of *Cytisus oromediterraneus* Rivas-Martinez *et al*. (‘piorno serrano’), although these species can enter the supramediterranean floor, reaching lower levels than is optimum. On the oromediterranean floor, these species include shrubs of timberlines and *Adenocarpus hispanicus* (Lam.) DC. or *Erica arborea* L. This vegetation cover alternates between grasslands, rocky outcrops and scree-talus. It is the most extensive vegetation in the Sierra de Guadarrama National Park [36], which forms the characteristic habitat of the summits of this mountain along with the areas covered by scree-talus (Fig 2). Finally, the crioromediterranean floor (2200–2400 m a. s. l.) has a combination of psychoxerophilic grasses and *Festuca curvifolia* Lag. *ex* Lange, which plays an important role in soil protection in extremely harsh climatic conditions due to its coverage [44]. In addition, there is an entire cohort of colluvial specialist vegetation [36] as well as large colluvial and morrenic deposits that extend along the oromediterranean floor.

In the Sierra de Guadarrama, the climate is a cold continental Mediterranean with dry and fresh summers. Its marked continentality due to its remoteness from the influence of the Mediterranean Sea and the Atlantic Ocean is not incompatible with the presence of a remarkable variety of microclimates. The microclimatic differences result from the special arrangement of the three mountain ranges, which condition the circulation of the air masses [45]. In addition, the topographic and geographical diversity (orientations, slopes, altitude) of each of these mountains decisively influence meteorological parameters, such as temperature, insolation, precipitation and the accumulation and persistence of snow [46]. The cold period extends beyond winter for about six months, while the dry period does not last longer than one month. The remaining five months are characterised by intense biological activity due to a combination of temperature and humidity that promotes the vegetative period of the plants [47]. Meteorological data of the port of Navacerrada (1894 m a. s. l.) recorded over a 20-year period yielded the following average annual values [48] that can guide the tendency of the main climatological variables of the Sierra de Guadarrama: maximum temperature 10.7°C; minimum temperature 3.1°C; 142 days of frost; and 1223 mm of precipitation over 111.3 days, of which 78 days was snow.

### Sampling

The sampling was performed mainly using Subterranean Sampling Devices (SSD) similar to those used in other underground surveys in Iberian enclaves [12, 25, 23, 49, 50, 51, 14]. The SSDs consisted of a PVC cylinder 11 cm in diameter and 1 m in length, which had numerous perforations that were 8 mm in diameter in its lower half (Fig 2F). Once buried vertically in a suitable substrate, the top was covered with the ground surface (Fig 2C–2E). A pitfall trap was slid inside using a nylon thread that precisely matched the tube wide (Fig 2G). Previously, the trap was partially filled with a preservative liquid (1,2-propanediol) and fed with a vial containing strongly odorous cheese (Fig 2G and 2I). Inside some SSDs (SSD-5, SSD-7, SSD-12, SSD-14, SSD-17 and SSD-23), a digital thermometer programmed to record temperature at one-hour intervals (Fig 2G) was installed and was protected by a plastic bell. In the La Mujer Muerta range, SSDs of 0.5 m perforated along their entire length except for the upper 20 cm were installed (Fig 2H) along with the four 1 m SSDs (SSD-1, SSD-2, SSD-3 and SSD-4) (Fig 2F). The purpose of the SSD model of 0.5 m was to collect the fauna of the most superficial MSS and thus allow for comparing the fauna collected with that extracted from the SSD of 1 m. Two pitfall traps (TSPs), which contained the same type of preservative and bait as the SSDs, were buried in a visible Colluvial MSS in a forest talus of the Puerto de Navafría. To achieve this, two horizontal holes of about 80 cm in length were created (Table 1). The coordinates used are Universal Transverse Mercator coordinate system (UTM).

**Table 1:** Location of the traps.

Mountains areas of the Sierra de Guadarrama	Code	Depth (m)	UTM Coordinates (100x100m)	Altitude (m a. s. l.)	Toponymy / Province	Date installation of Traps	Date of trap recovery	Orientation
Siete Picos—La Mujer Muerta	SSD-1	1	30 T 4081 45204	1606	Cancho del Río Peces / Segovia	20/05/2015	17/09/2015	North
	SSD-1 (0.5)	0.5						
	SSD-2	1	30 T 4100 45166	1818	Corrales de la Majada Minguete / Segovia	20/05/2015	17/09/2015	Northeast
	SSD-2 (0.5)	0.5						
	SSD-3	1	30 T 4068 45192	1622	Umbría de la Mujer Muerta / Segovia	21/05/2015	17/09/2015	North
	SSD-3 (0.5)	0.5						
	SSD-4	1	30 T 4056 45181	1685	Majada Conejo / Segovia	21/05/2015	17/09/2015	Northwest
	SSD-4 (0.5)	0.5						
	SSD-11	1	30 T 4108 45161	1876	Cerro Ventoso / Madrid	09/06/2015	17/09/2015	East
Puerto de los Cotos—Puerto de Navacerrada	SSD-5	1	30 T 4166 45159	1923	Arroyo Seco / Segovia	27/05/2015	22/09/2015	Northwest
	SSD-6	1	30 T 4179 45185	1787	La Pedriza / Segovia	27/05/2015	22/09/2015	Northwest
Montes Carpetanos	SSD-7	1	30 T 4185 45229	1994	Majada Hambrienta / Segovia	02/06/2015	17/09/2015	Northwest
	SSD-8	1	30 T 4190 45231	2071	Majada Aranguez / Segovia	02/06/2015	17/09/2015	Northwest
	SSD-9	1	30 T 4187 45211	2208	Dos Hermanas / Madrid	03/06/2015	05/10/2015	East
	SSD-10	1	30 T 4191 45213	2049	Hoya de la Laguna Grande / Madrid	03/06/2015	05/10/2015	East
	SSD-16	1	30 T 4334 45389	1956	Las Revueltas—Los Horcos / Segovia	23/06/2015	07/10/2015	West
	SSD-17	1	30 T 4347 45414	1976	Peña del Buitre / Segovia	23/06/2015	07/10/2015	Northwest
	SSD-18	1	30 T 4373 45438	1885	Los Loberos / Segovia	23/06/2015	07/10/2015	Northwest
	SSD-19	1	30 T 4224 45307	1866	La Gelecha—La Flecha / Madrid	24/06/2015	06/10/2015	Southeast
	SSD-20	1	30 T 4226 45332	1937	Cerro de Navahonda / Segovia	24/06/2015	06/10/2015	Northeast
	SSD-21	1	30 T 4211 45274	1891	El Paredón / Madrid	24/06/2015	06/10/2015	Northeast
	SSD-22	1	30 T 4304 45376	1995	Alto del Puerto / Segovia	24/06/2015	22/09/2015	North
	SSD-23	1	30 T 4288 45367	2144	Circo del Pico Nevero / Madrid	25/06/2015	06/10/2015	Sourtheast
	SSD-24	1	30 T 4274 45357	2042	Peñacabra / Madrid	25/06/2015	22/10/2015	East
	SSD-25	1	30 T 4249 45407	1731	Arroyo del Charco (La Cepa) / Segovia	02/07/2015	22/10/2015	Northwest
	TSP-1	0.8	30 T 4314 45376	1780	Puerto de Navafría / Segovia	24/06/2015	22/09/2015	North
	TSP-2	0.8	30 T 4314 45376	1780	Puerto de Navafría / Segovia	24/06/2015	22/09/2015	North
Cuerda Larga and Associated Mountainous complex	SSD-12	1	30 T 4180 45138	2102	Collado del Piornal / Madrid	09/06/2015	22/09/2015	North
	SSD-13	1	30 T 4179 45135	2113	Los Almorchones—Las Buitreras / Madrid	10/06/2015	22/09/2015	Southwest
	SSD-14	1	30 T 4274 45224	1406	El Purgatorio / Madrid	18/06/2015	05/10/2015	West
	SSD-15	1	30 T 4273 45224	1375	Hueco de los Ángeles / Madrid	18/06/2015	05/10/2015	Northeast
	SSD-26	1	30 T 4309 45186	1890	La Najarra—Cuatro Calles / Madrid	02/07/2015	30/10/2015	East
	SSD-27	1	30 T 4270 45185	2101	Bailaderos / Madrid	02/07/2015	30/10/2015	North
	SSD-28	1	30 T 4193 45164	2156	Collado de Valdemartín / Madrid	03/07/2015	06/11/2015	North
	SSD-29	1	30 T 4211 45168	2301	Cabeza de Hierro Mayor Menor / Madrid	03/07/2015	06/11/2015	Crest
	SSD-30	1	30 T 4227 45170	2233	Collado de Peña Vaqueros (Loma de Pandasco) / Madrid	03/07/2015	06/11/2015	Crest
	SSD-31	1	30 T 4288 45184	1946	Collado de la Najarra / Madrid	09/07/2015	22/10/2015	North
	SSD-32	1	30 T 4285 45187	1948	Arroyo de La Najarra / Madrid	09/07/2015	22/10/2015	Northeast
	SSD-33	1	30 T 4286 45188	1819	Arroyo de La Najarra / Madrid	09/07/2015	22/10/2015	North

(SSD, Subterranean Sampling Device; TST, Talus slope pitfall).

The enclaves sampled were located within the national park (32 localities), and to a lesser extent, within the area declared Peripheral Protection Area (2 localities) (Table 1, Fig 1): a) five locations in Siete Picos and La Mujer Muerta, of which four had double SSDs (1 m and 0.5 m); b) 15 localities in Montes Carpetanos, one of which had two TSPs installed and the remaining 14 an SSD of 1 m in length; and c) 12 localities with SSDs of 1 m in length, located in Cuerda Larga and its associated mountainous complex (eight in Cuerda Larga, two in La Maliciosa, the highest area of the Sierra de los Porrones, and two in the Altos de la Morcuera). The authors of the sampling included a team that consisted of Ortuño V.M., Ledesma E., Gilgado J.D., Jiménez-Valverde A., Pérez-Suárez G. and Baquero E.

Sampling permits for the corresponding national parks were obtained from the following appropriate authorities: Ismael Hernández Fernández, Deputy Director General of Management and Planning of Protected Areas (2015) and José Lara Zabía, Head of Conservation Area of Flora and Fauna (2016) at the General Directorate of Environment of the Community of Madrid, and Jose Ignacio Quintanilla Rubio (2015), and Montserrat de Andrés Boal (2016), General Director of the Natural Environment by delegation of the Head of the Territorial Service of the Environment of the Junta de Castilla y León.

The biological samples (specimens collected from May to October 2015) were transported to the laboratory and were preserved in 70% ethanol until the taxonomic study began.

### Mounting and observation

Some specimens were cleared in Nesbitt’s fluid, and after washing for one hour in 70% Ethyl alcohol, they were mounted in Hoyer’s medium for the compound microscope observation in a phase contrast and DIC. Some specimens were decapitated, immersed in a saturated solution of KOH and emptied before being mounted on the slides.

Some specimens were examined using a Scanning Electron Microscope (SEM). Specimens from 70% ethyl alcohol were slowly rehydrated in a decreasing series of concentrations (60%, 50%, 40%, 30%, 20%, 10%, distilled water) over 24 hours. Once in distilled water, they were fixed in 4% glutaraldehyde in a cacodylate buffer for 24 h and then transferred to sucrose 0.25 M for 24 h. The specimens were then dehydrated up to 100% ethyl alcohol. Complete desiccation was achieved using the CO_2_ critical point technique. Samples were then covered with a 16-nm thin layer of molecular gold using an Emitech K550 sputter coater. Observations were performed using a Zeiss Digital Scanning Microscope 940 A.

### Taxonomy

The combined use of color and morphological characters (macrochaetotaxy included) allowed for the identification of the species and provided useful descriptions for species identification. Potapov and Kremenitsa used coloration and macrochaetotaxy (some selected areas), the antennae length, width and shape, the eye structure, leg length, claw complex and manubrial organ (males) [33]. The array (or group) of characteristics proposed by Jordana and Baquero [32] and the chaetotaxy nomenclature of [31], which is based on a constant and generally visible set of morphological characters [52, 53, 54], has proven useful for the identification of species that belong to Entomobryini. It is proposed that some of the areas that conform to the simplified formula of [32] for *Entomobrya* could also apply to *Orchesella*, and an area for the head macrochaetotaxy (H6) has been added, which indicates the presence of chaetae S_0_ and S_2_ and their accessories.

## Results

### Fauna associated with the Colluvial MSS

For the identification of the Collembola species examined in the present study, a total of 42,240 specimens (20% belonging to the genus *Orchesella*) were studied individually. This work was conducted at the Department of Environmental Biology, University of Navarra, after the triage was completed at the University of Alcalá for over 61,038 specimens. This means that 69 per cent of the specimens caught in the total traps belonged to the Collembola taxon.

Throughout the first collection of sampling traps from the MSS of the Sierra de Guadarrama, only for the fauna of Collembola, a total of 32 genera and 61 species were identified. Of these 61 species, 18 are likely new to science. The taxon Entomobryidae, with five genera and 18 species, was the most uniformly distributed throughout the entire study area. The genus *Orchesella*, represented by two new species, was present in almost all traps (absent only in four). Thus, these two species are considered the most representative of the Colluvial MSS of the Sierra de Guadarrama.

### Description of new species

Class **COLLEMBOLA** Lubbock, 1870

Order **ENTOMOBRYOMORPHA** Börner, 1913, sensu Soto-Adames *et al*., 2008

Family **ENTOMOBRYIDAE** Tömösvary, 1882

Subfamily **ORCHESELLINAE** Börner, 1906

Tribe **ORCHESELLINI** Börner, 1906

Genus ***Orchesella*** Templeton, 1836

**Fig 3:**
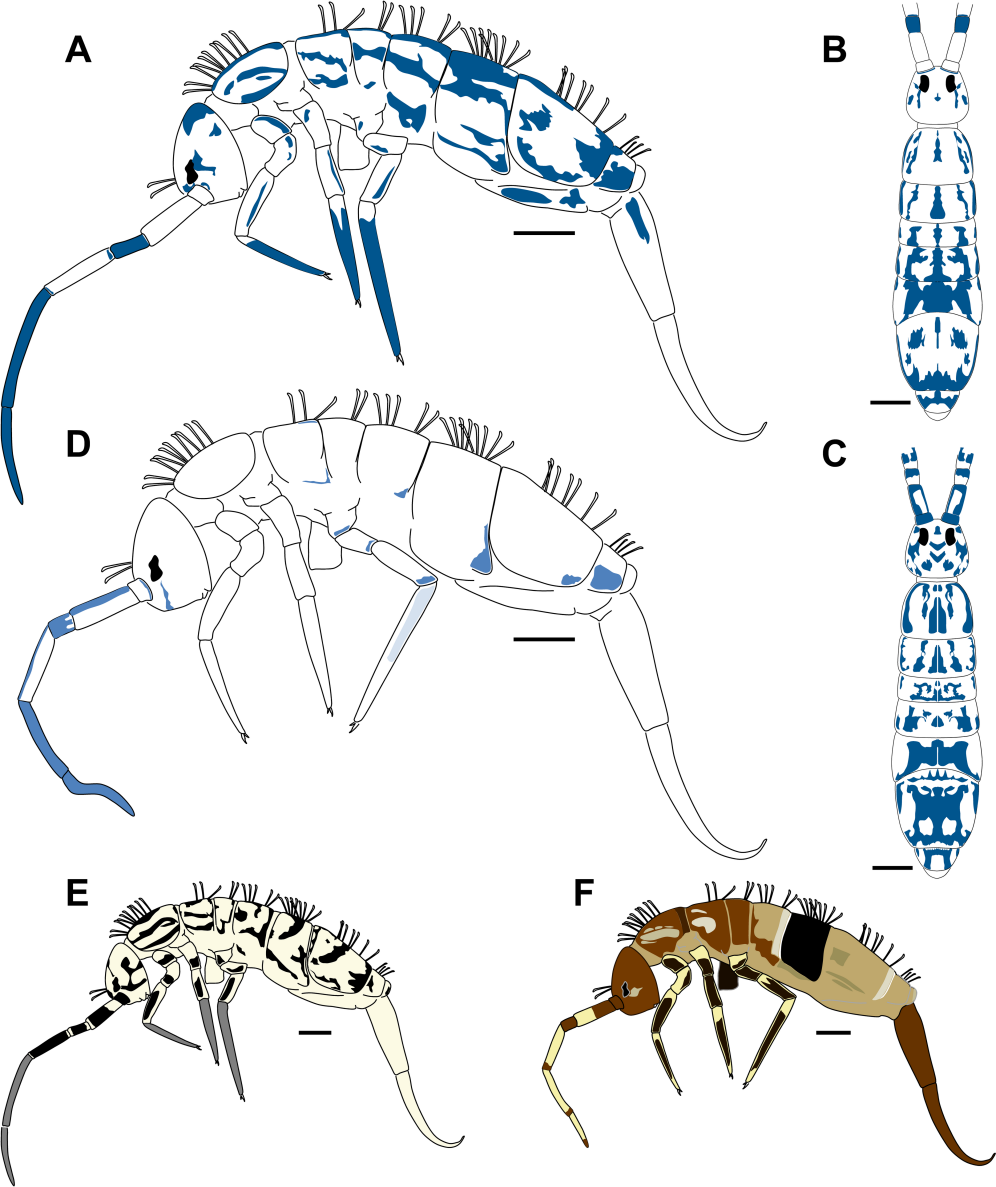
Habitus and color pattern of the *Orchesella* species studied. (A) *Orchesella mesovoides* sp. nov., lateral view. (B) *Orchesella mesovoides* sp. nov., dorsal view. (C) *Orchesella eolia*, dorsal view. (D) *Orchesella colluvialis* sp. nov., lateral view. (E) *Orchesella villosa*, lateral view. (F) *Orchesella cincta*, lateral view (bar 0.5 mm).

**Fig 4:**
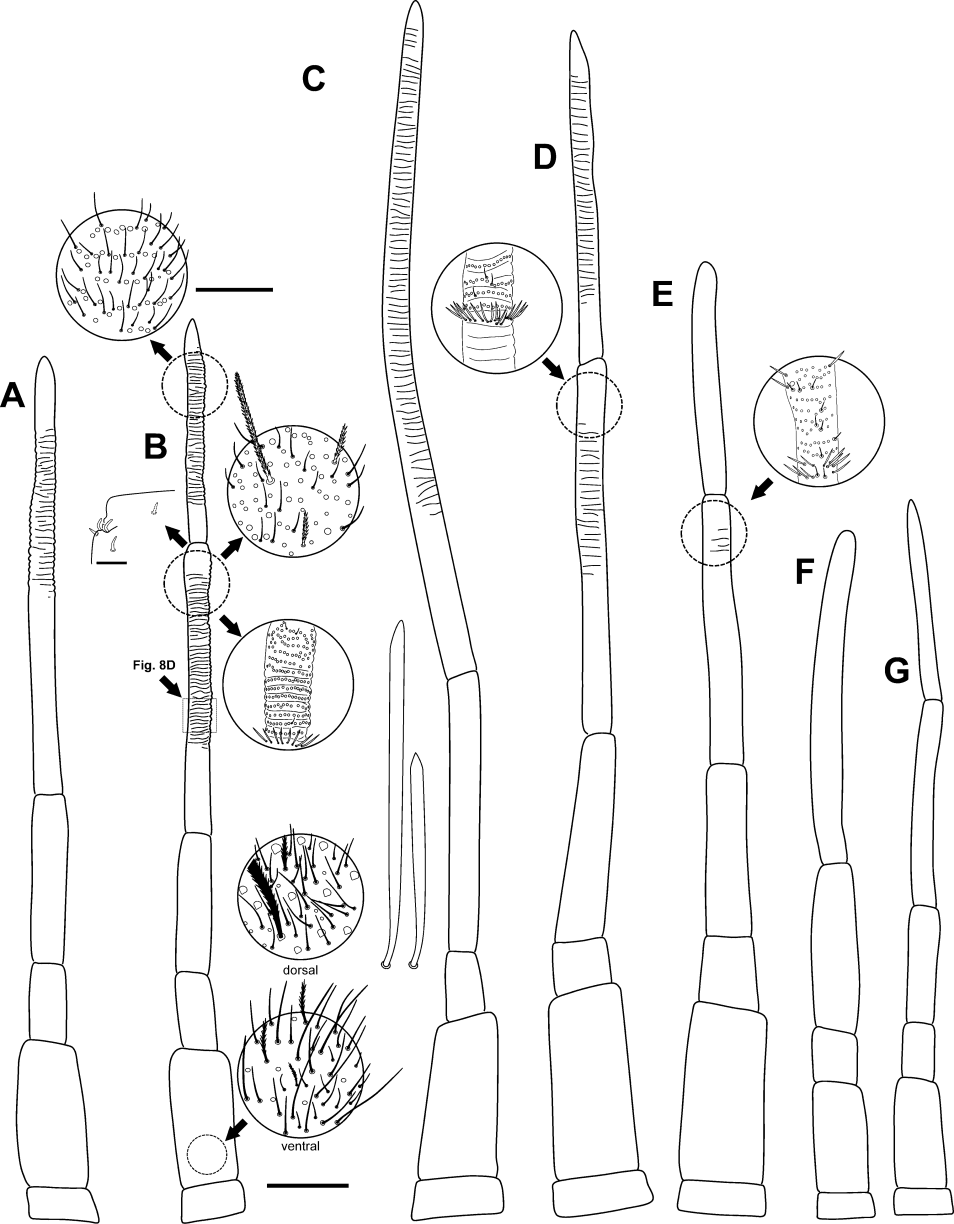
Antennae of the *Orchesella* species studied. (A–B) *Orchesella mesovoides* sp. nov. aberrant antenna, with a *flagellus* instead of the normal third and fourth segments and a normal antenna with some areas enlarged. (C–D) *Orchesella colluvialis* sp. nov. antenna. (E), *Orchesella villosa*. (F–G) *Orchesella cincta* (bar for antennae: 0.25 mm; bar for enlarged areas: 0.05 mm).

**Fig 5:**
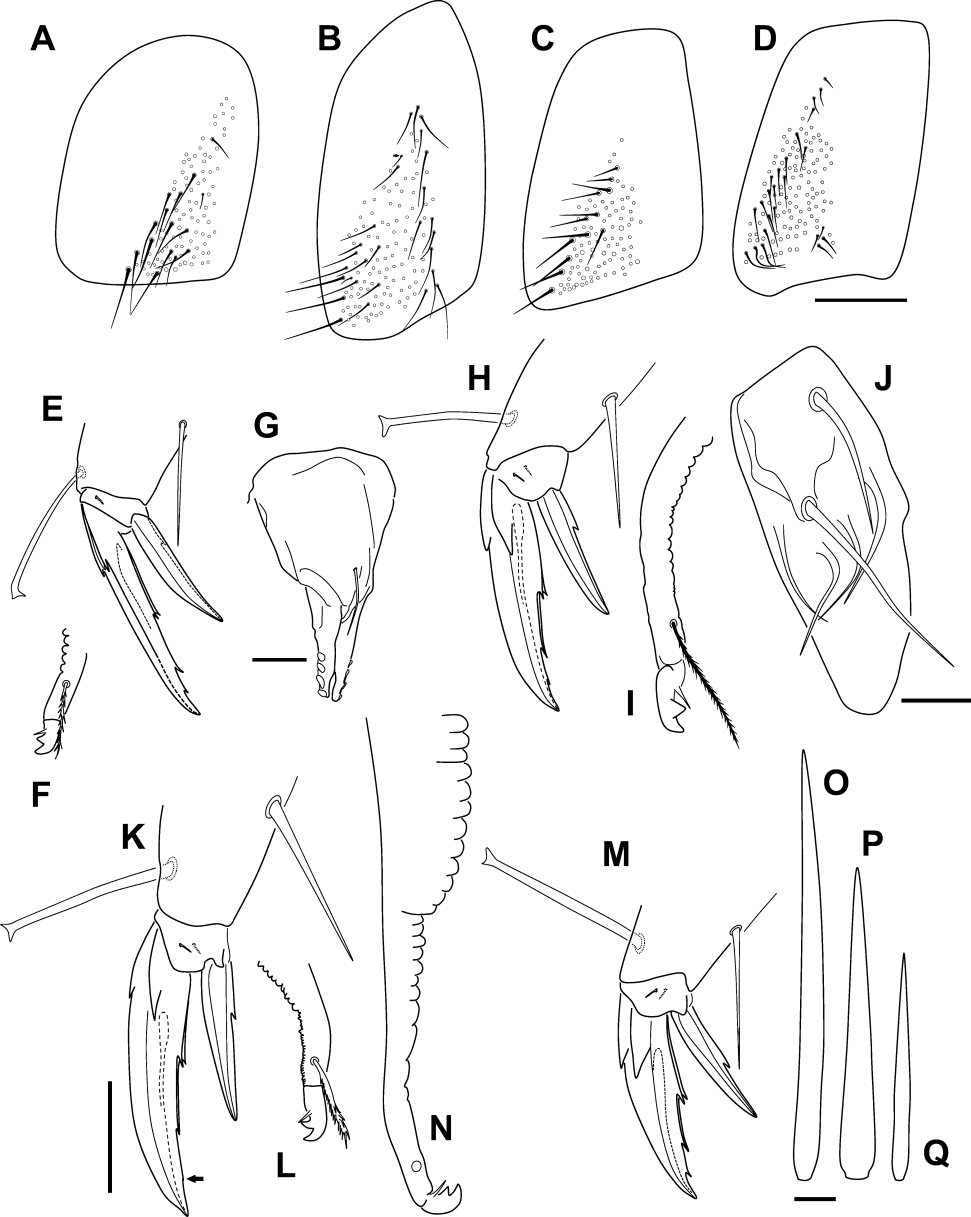
Some morphological characters of the *Orchesella* species studied. (A) Trochanteral organ of *Orchesella mesovoides* sp. nov. (B) Trochanteral organ of *Orchesella colluvialis* sp. nov. (C) Trochanteral organ of *Orchesella villosa*. (D) Trochanteral organ of *Orchesella cincta*. (E) Tip of the leg III (claw and empodial appendage, anterior view) of *Orchesella mesovoides* sp. nov. (F) Tip of the dens and mucro of *Orchesella mesovoides* sp. nov. (G) Tenaculum of *Orchesella mesovoides* sp. nov. (H) Tip of the leg III of *Orchesella colluvialis* sp. nov. (I) Tip of the dens and mucro of *Orchesella colluvialis* sp. nov. (J) Maxilar outer lobe of *Orchesella colluvialis* sp. nov. (K), Tip of the leg III of *Orchesella villosa* (the arrow points to the absent tooth in the drawn claw). (L) Tip of the dens and mucro of *Orchesella villosa*. (M) Tip of the leg III of *Orchesella cincta*. (N) Tip of the dens and mucro of *Orchesella cincta*. (O–Q) Macrochaetae from tibiotarsus of leg III of the *Orchesella colluvialis* sp. nov.: long, special and short (normal size), respectively (bars: A–B: 0.1 mm; E–F, H–I, K–L, M–N: 0.5 mm; G: 0.04 mm; J, O–Q: 0.02 mm).

**Fig 6:**
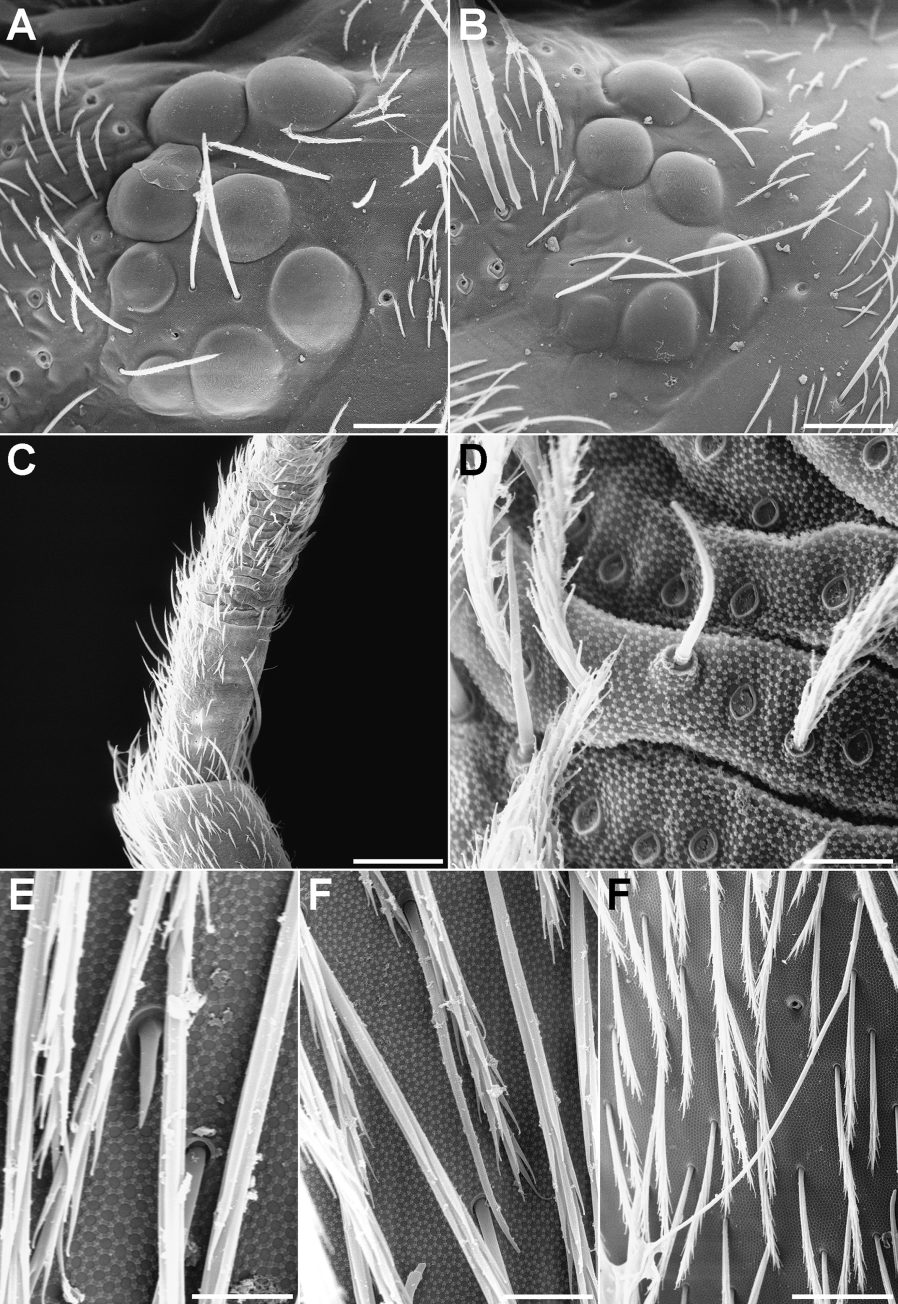
SEM photographs. (A) Eye of the *Orchesella mesovoides* sp. nov. (bar 0.04 mm). (B) Eye of the *Orchesella colluvialis* sp. nov. (bar 0.04 mm). (C) Tip of the antennal segment II and the basal part of III of the *Orchesella mesovoides* sp. nov. (bar 0.09 mm). (D) Detail of the antennal segment III in an area with apparent segmentation of the *Orchesella mesovoides* sp. nov. (bar 0.006 mm). (E) S-microchaeta from the Abd III of the *Orchesella colluvialis* sp. nov. (bar 0.004 mm). (F) S-chaeta from the Abd II of the *Orchesella mesovoides* sp. nov. (bar 0.006 mm). (G) S-chaeta from the Abd IV of the *Orchesella mesovoides* sp. nov. (bar 0.02 mm).

**Fig 7:**
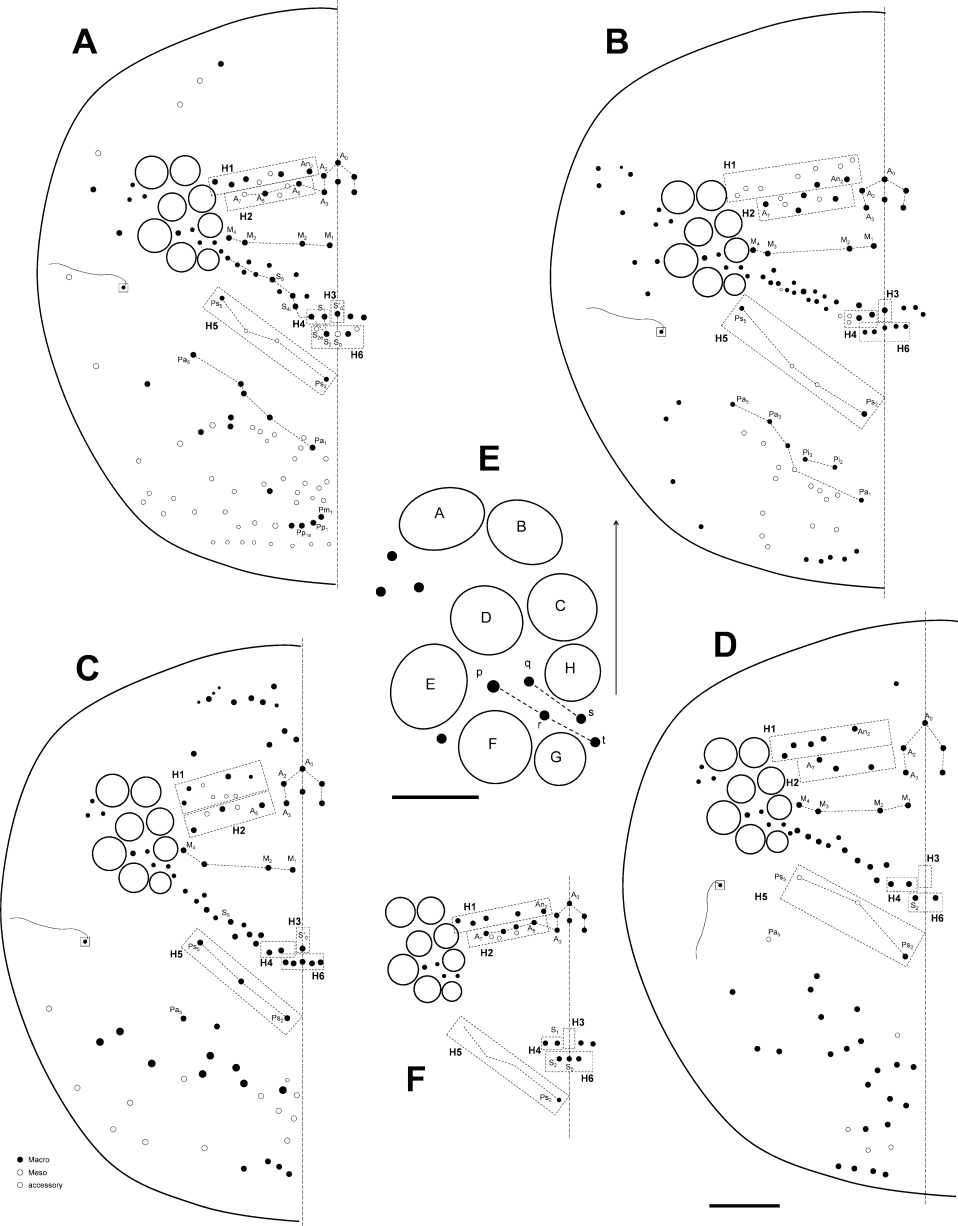
Head chaetotaxy of the *Orchesella* species studied. (A) *Orchesella mesovoides* sp. nov.; (B) *Orchesella colluvialis* sp. nov. (C) *Orchesella villosa*. (D) *Orchesella cincta*. (E) Eye details of the *Orchesella mesovoides* sp. nov. (F) Main chaetotaxy of *Orchesella eolia* (bar 0.1 mm except for E, 0.04 mm).

**Fig 8:**
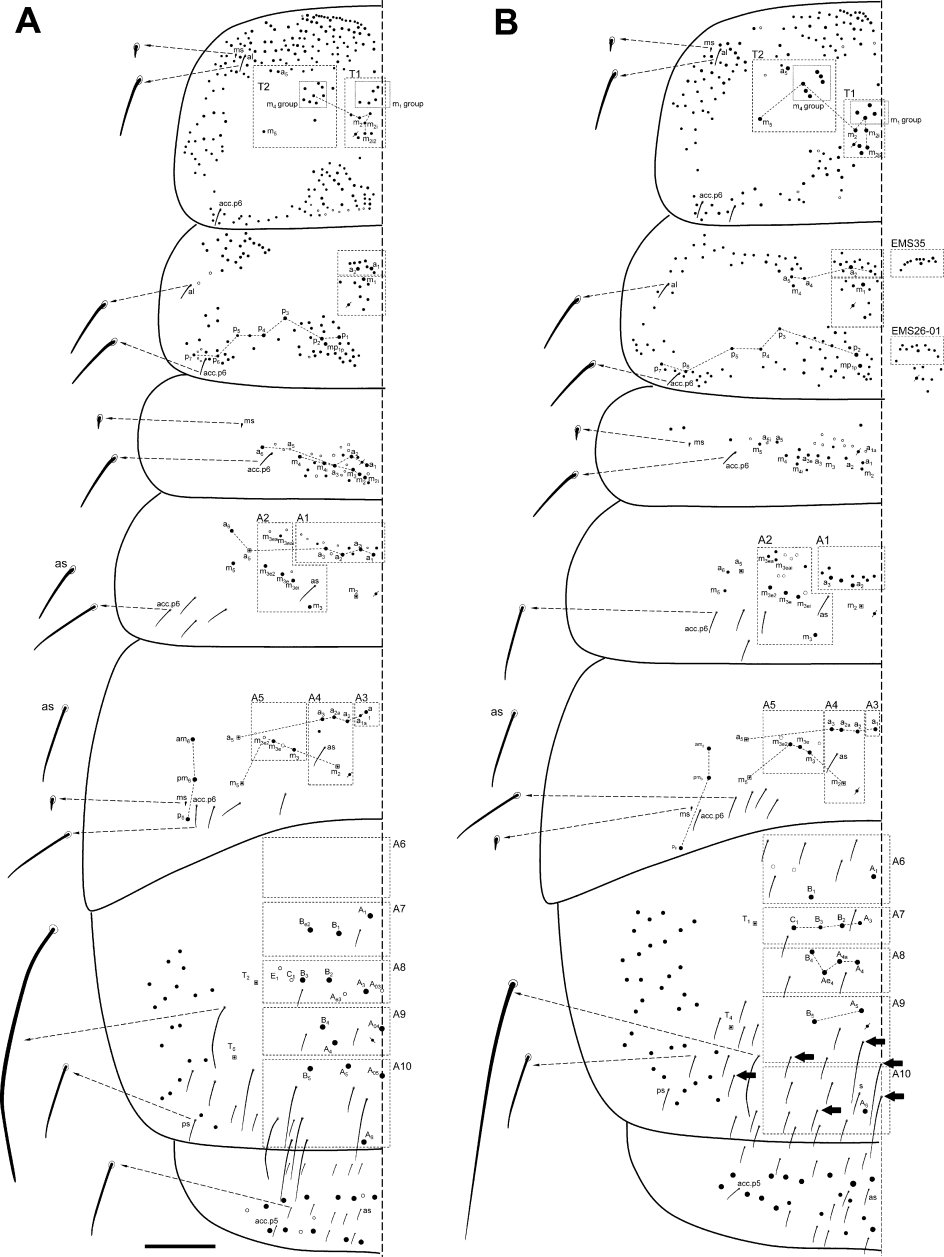
Dorsal macrochaetotaxy of Th II to Abd V. (A) *Orchesella mesovoides* sp. nov. (B) *Orchesella colluvialis* sp. nov. (bar 0.2 mm; S-chaetae and S-microchaetae drawing in the lateral x10).

***Orchesella mesovoides*** Baquero and Jordana sp. nov.

urn:lsid:zoobank.org:act:BB75F525-25F4-4C47-AABD-EDFC132141B8

(Figs 3A, 3B, 4A, 4B, 5A, 5E–5G, 6A, 6C, 6D, 6F, 6G, 7A, 7E and 8A)

Type material.—**Holotype**: female, trap SSD-2 (slide 23), Corrales de la Majada Minguete (30 T 410083 4516653, 1818 m a.s.l.), Mujer Muerta/Montón de Trigo, Sierra de Guadarrama, Segovia, SPAIN, 17.IX.2015, pitfall SSD (since 20.V.2015), Ortuño, *et al*. leg. **Paratypes**: SSD-1, 26 in ethanol; SSD-2, 6 in slides (22, 1 female; 24, 1 juvenile; 25, 1 female; 26, 1 male; 27, 1 female), 582 in ethanol; SSD-3, 29 in ethanol; SSD-4, 8 in ethanol (all from La Mujer Muerta/Montón de Trigo, Sierra de Guadarrama); SSD-5, 2 in ethanol; SSD-7, 116 in ethanol; SSD-8, 4 in ethanol; SSD-9, 50 in ethanol; SSD-11, 123 in ethanol; SSD-12, 184 in ethanol; SSD-13, 2 in ethanol; SSD-15, 6 in ethanol; SSD-16, 100 in ethanol; SSD-17, 20 in ethanol; SSD-18, 3 in ethanol; SSD-21, 120 in ethanol; SSD-22, 206 in ethanol; SSD-25, 35 in ethanol; SSD-26, 1 male in slide, 1 in ethanol; SSD-29, 4 in ethanol; SSD-30, 252 in ethanol; SSD-31, 20 in ethanol; SSD-33, 50 in ethanol; TSP-1, 12 in slides (01, 3 males; 02, 2 males; 03, 1 female; 04, 1 male; 05, 1 male; 06, 1 male; 07, 3 males); TSP-2, 1 male on slide (01).

Other material studied.—Collado Alto (Alto del León), mina de wolframio, Coordinates: 40.68660815, -4.16397343, 1 specimen labeled *Orchesella eolia*, Museo Nacional de Ciencias Naturales, Madrid (MNCN)_Ent 144262; Valsaín, 26-VI-63, 1 specimen labeled *Orchesella eolia*, MNCN_Ent 144266 (MNCN).

Etymology.—The species name follows the ecological niche. This was the first time it had been studied in Sierra de Guadarrama, where it was found.

All specimens were deposited in Museum of Zoology, University of Navarra, Pamplona, Spain (MZNA) except two paratype slides: TSP-1 01, three males, in MNCN; TSP-1 02, two males, in Muséum d’histoire naturelle de Genève (MHNG).

#### Description

Body length 3.70–5.80 mm (mean 4.36 mm, n = 17) (Holotype 5.77 mm) (Fig 3A and 3B).

Ground color of the body pale; eyes black; head with light blue pigment on lateral and posterior head; antennae slightly blue at the end of the second part of the first segment, dark blue in the first part of the second segment and slightly blue for the last two segments; Thorax (Th) II–Abdomen (Abd) II with lateral, dorsolateral and dorsal-central longitudinal dark blue stripes, broadened at the end of each segment; Abd III with a transversal dark blue irregular band; Abd IV characterised by a continuation of the pigment patches of the forwarded segments; Abd V with dorsal and lateral spots; Abd VI without pigment; legs: femur with terminal lateral blue pigment and tibiotarsus homogenously light blue. Juveniles with a similar coloration.

Head. Ratio antennae/head = 4.04 (n = 14). First and second sub-segments of Antennal segment (Ant) I thick, twice as Ant II. Distal part of Ant III and Ant IV annulated and verticillate (Figs 4A and 4B and 6C and 6D), without apical bulb; Ant III sensory organ as in Fig 4B (detail). Labral papillae rounded, with pointed and curved tip, as a hook. Prelabral and labral chaetae all smooth (4/554). Lateral process (or differentiated chaeta) of outer labial palp (E) not reaching the middle of its papilla. Anterior labial row with four smooth and subequal chaetae; posterior labial row internal to chaeta E with 6–8 chaetae per side, all ciliated; chaetae E, L_1_ and L_2_ ciliated. Outer lobe of maxillary palp with four smooth chaetae in addition to the two basal chaetae. 8 + 8 ommatidia, G and H smaller (Fig 6A).

Body. Abd IV/III ratio 1.40–2.25 (mean 1.75, n = 17). Trochanteral organ with approximately 100 special chaetae (Fig 5A). Tibiotarsus bent distally, as a sub-segmentation, at 55% of its total length; spiniform chaetae with appressed ciliation (apparently wide, smooth chaetae): 7–10 on leg 1; 10–15 on legs 2 and 3. Claw with one outer tooth, a little more distal than a pair of stout lateral teeth, and four inner teeth (two paired at 50%, a first unpaired at 75% and a minute pair at 90% of the internal edge’s total length). Empodial appendage lanceolated and concave, with the interior (towards claw) very curved and with an outer tooth inserted at 30–40% of its total empodial length; empodial appendage/claw length ratio 0.62 (0.57–0.65, n = 7); smooth chaetae on tibiotarsus similar in length to empodial appendage (0.84–1.09, n = 7); tenent hair similar in length to empodial appendage (n = 7) (Fig 5E). Anterior face of ventral tube with many chaetae (>50), including 2+2 Mc on distal area; posterior face with >50 chaetae, including 1+1 Mc on latero-basal area; apical flaps with >50 smooth and ciliate chaetae. Tenaculum as in Fig 5G.

Furcula. Males with manubrial organs with four small chaetae grouped similar to a brush. The smooth area of distal dens 4 to 5 times the mucronal length; basal spine on mucro reaching or slightly surpassing the basal tooth; teeth of similar size (Fig 5F).

Macro-chaetotaxy (Figs 7A, 7E and 8A, Table 2). Head: H1 area with five to seven An chaetae, some as mc (mesochaetae); H2 area with 2–3 Mc (Macrochaetae) and 2–3 mc; H3 area with S’_0_ present; H4 area with two Mc; the S series (from S_4i_ to the eye) with 10–13 chaetae, 3–4 as Mc (chaetae of different sizes); H5 area with two Mc; H6 area with four or five Mc (sometimes as mc). Th II: T1 area with 10–12 Mc (n = 4); T2 area with 7–10 Mc, m_5_ not seen in one of the four specimens studied. Th III with three Mc above pseudoporus, and 12–13 chaetae in total; lateral sensillae not identified. Abd I with five Mc around the pseudoporus (a_1_, a_2_, m_2i_, m_2_ and m_3_), and four towards lateral (a_3_, m_4i_, m_4_ and a_6_); lateral sensillae not identified. Abd II with four Mc in A1 area (a_1_, a_2i_, a_2_ and a_3_), and four on A2 area (m_3_, m_3ei_, m_3e_ and m_3e2_); sensilla ‘as’ above m_3_ (between m_3_ and a_3_); m_2_ and a_5_ as bothriotricha. Abd III with one Mc on A3 area (a_1_), three on A4 area (a_2_, a_2a_ and a_3_) and two on A5 area (m_3_ and m_3e_); sensilla ‘as’ above m_2_ (between m_3_ and a_3_); a_5_, m_2_ and a_5_ as bothriotricha, three Mc lateral to external bothriotricha. Abd IV: A6 area without Mc; A7 area with three Mc (A_1_, B_1_ and B_e2_); A8 area with three Mc (A_3_, B_2_ and B_3_) and four mc (A_03_, A_e3_, C_1_ and E_1_); A9 area–than included the *pseudopore*, in a more advanced position than in other species of the family that were closer to A_5_– with three Mc (unpaired A_04_, A_4_ and B_4_); and A10 area with four Mc (unpaired A_05_, A_5_, B_5_ and A_6_); and A10 with only one Mc (A_6_).

Simplified Mc formula: 5(7)-2(5)-1-2(3)-2-4(5)/12-9/4-4/1-3-2/0-3-1_0_3(6)-1_0_2-1_0_3 (1_0_ is an unpaired chaeta; maximum in parentheses).

**Table 2:** Comparison of characters among the different species of Orchesella cited in this paper.

	1	2	3	4	5	6	7	8	9	10	11	12	13	14	15	16	17	18	19	20	21	22
***O*.* cincta***	5	3	0	2	1	2	7	7	4	4	3	1	2	3	0	3	0	1	0	0	0	0
***O*.* colluvialis* sp. nov.**	2(9)	4(7)	1	2(3)	2	5	7(9)	8	4	3	4	1	3	3	2	4	0	4	0	2	0	1
***O*.* eolia***	5	4(7)	0	2	1	3	11	8	4	4	7(8)	0	2	4(5)	0	7	0	2	0	2	0	0
***O*.* mesovoides* sp. nov.**	5(7)	2(5)	1	2(3)	2	4(5)	12	9	4	4	4	1	3	2	0	3	1	3(6)	1	2(3)	1	3
***O*.* villosa***	4(8)	3(6)	1	2	3	5	7	5	3(4)	6	5	0(1)	3	3	0	2	0	3	0	0	0	0

(1), H1 area (head): Mc on series sd'4-sd'4' (An2-An3), total number; (2), H2 area (head): Mc on series sd4-sd'3a (A5-A7), total number; (3), H3 area (head): Mc on series d'0 (S'0), total number; (4), H4 area (head): Mc on series d1-sd1-sd'1 (S1-S3-S4), total number; (5), H5 area (head): Mc on series v1-v3-v4 (Ps2-Ps3-Ps5), total number; (6), H6 area: Mc on series S0-S2 and accessory, total number; (7), T1 area (th II): Mc on series m1-m2i2; total number; 5 if >4; (8), T2 area (th II): Mc on series a5-m5; total number; 9 if >8; (9), claw, internal teeth: total number; (10), A1 area (Abd II): Mc on series a2-a3, total number; (11), A2 area (Abd II): Mc on m3 series, total number; (12), A3 area (Abd III): Mc on series a1, total number; (13), A4 area (Abd III): Mc on series above m2, total number; (14), A5 area (Abd III): Mc on m3-m4 series, total number; (15), A6 area (Abd IV): Mc on series a1-a5 (A1-E1a), total number; 9 if >8; (16), A7 area (Abd IV): Mc on series ma1-ma4 (A2-E1), total number; 10 if >9; (17), A8 area (Abd IV), unpaired chaeta: Mc on series m0 (A04), total number; (18), A8 area (Abd IV): Mc on series m1-m3 (A4p-C4), total number; 6 if >5; (19), A9 area (Abd IV), unpaired seta: Mc on series mp0 (A05), total number; (20), A9 area (Abd IV): Mc on series mp1-mp3 (A5-B5), total number; 6 if >5; (21), A9 area (Abd IV), lateral: Mc, total number; (22), A10 area (Abd IV): Mc on series p1a-p3 (A6i-B6), total number. In parenthesis, maximum number of chaetae (Mc or mc).

S-chaetae formula: 2, 2/1, 5, 5, n, 13 (n = approximately 10–18, some of them four times the ‘normal’ (short) S-chaetae). The ‘normal’ S-chaetae (Fig 6F) measured 24–26 micrometers, while the ‘long’ S-chaetae on Abd IV (Fig 6G) measured 95–100 micrometers. S-microchaetae formula (like spine, approximately 3.5 micrometers): 1, 0/1, 0, 1, 0, 0 (Fig 8A).

#### Biology

The study of the intestinal content of the specimens mounted on the slides allowed for determining that they feed mainly on amorphous organic matter. In addition, in almost every specimen, fungal hyphae and spores were observed. There was also a noticeable quantity of crystals (presumably small sand grains).

#### Remarks

The coloration of the species *O*. *mesovoides* sp. nov was completely different from the seven species mentioned in the Iberian Peninsula, though it was somewhat similar to the original description of *O*. *eolia* (from Stromboli Island, cited from the Sierra de Guadarrama) [55]. Two of these specimens cited by Selga were observed. The first was from Collado de la Mina (or Lijar) in Alto del León (mine of wolframio) (MNCN_Ent 144262). Another specimen from Sierra de Navacerrada was a juvenile that was cleared and mounted sideways and the chaetotaxy is not visible, (MNCN_Ent 144270). The first belonged to the *O*. *mesovoides* sp. nov. due to its coloration with a less extended pigment, and the macro-chaetotaxy was also similar.

It was possible to identify differences in the pigment patterns between the new species and *O*. *eolia*, especially in the Th II and Abd II-V segments. The ratio Abd IV/III in *O*. *eolia* (1.48) was lower than that for the species *O*. *mesovoides* sp. nov. (1.40–2.25). The macrochaetotaxy was considerably different between the species *O*. *mesovoides* (Figs 7A and 8A) and *O*. *eolia* (Fig 7F). *O*. *eolia* had more Mc in H2 area, and H3 area was present in *O*. *mesovoides* sp. nov. but was absent in *O*. *eolia* (Table 2).

***Orchesella colluvialis*** Jordana and Baquero sp. nov.

urn:lsid:zoobank.org:act:3881F811-7E6A-4776-8339-92EFCC34DF3D

(Figs 3D, 4C and 4D, 5B, 5H–5J, 5O–5Q, 6B, 6E, 7B and 8B)

Type material.—**Holotype**: female, trap SSD-22 (slide 01), Canchal del Alto del Puerto (30 T 430419 4537625, 1995 m), Montes Carpetanos, Sierra de Guadarrama, Madrid, SPAIN, 22.IX.2015, pitfall SSD (since 24.VI.2015), Ortuño *et al*. leg. **Paratypes**: SSD-1, 5 in slides (09, 1 female; 10, 1 male; 11, 1 female; 12, 1 female; 13, 1 juvenile;), 467 in ethanol; SSD-2, 187 in ethanol; SSD-3, 4 in slides (01, 1 female; 02, 1 juvenile; 03, 2 juvenile), 425 in ethanol; SSD-4, 279 in ethanol; SSD-5, 96 in ethanol; SSD-6, 389 in ethanol; SSD-7, 216 in ethanol; SSD-8, 168 in ethanol; SSD-9, 163 in ethanol; SSD-10, 10 in ethanol; SSD-11, 117 in ethanol; SSD-12, 115 in ethanol; SSD-13, 443 in ethanol; SSD-14, 40 in ethanol; SSD-15, 68 in ethanol; SSD-16, 201 in ethanol; SSD-17, 132 in ethanol; SSD-18, 75 in ethanol; SSD-19, 55 in ethanol; SSD-20, 57 in ethanol; SSD-22, 184 in ethanol; SSD-25, 173 in ethanol; SSD-26, 1 female in slide (01), 591 in ethanol; SSD-27, 112 in ethanol; SSD-28, 38 in ethanol; SSD-29, 135 in ethanol; SSD-30, 73 in ethanol; SSD-31, 700 in ethanol; SSD-33, 1 female in slide (01), 220 in ethanol.

Etymology.—The species name follows the ecological niche of where it was found (rock detritus and soil accumulated at the foot of a mountain) from Latin *colluvium*.

All specimens were deposited in MZNA except two paratypes: SSD-1 11a, female, in MNCN; SSD-26 01, male, in MHNG.

#### Description

Body length 4.50–6.40 mm (mean 5.50 mm, n = 13) (Holotype 4.53 mm) (Fig 3D).

Ground color of the body uniformly pale; eyes black; head with light blue pigment only lateral to eyes and insertion of Ant I; antennae pigmented on dorsal Ant I b, Ant II a, dorsal Ant II b, and last two segments; only the lateral Th III, Abd II–III, posterior lateral Abd IV, lateral Abd V, and in some specimens, external distal trochanter, femur and tibiotarus, had some pigment.

Head. Ratio antennae/head = 4.30 (n = 11). First and second sub-segments of Ant I thickness, twice that of Ant II. Distal part of Ant III and Ant IV annulated and verticillate (Fig 4C and 4D) without apical bulb; Ant III sensory organ similar to that of *O*. *mesovoides* sp. nov. Labral papillae rounded, with pointed and curved tip, as a hook; in some specimens hook asymmetrically bifurcate, sometimes with a bristle, with one to three ramus. Prelabral and labral chaetae all smooth (4/554). Lateral process (or differentiated chaeta) of outer labial palp (E) not reaching the middle of its papilla. Anterior labial row with four smooth and subequal chaetae; posterior labial row internal to chaeta E with 13–15 chaetae per side, all ciliated; chaetae E, L_1_ and L_2_ ciliated (E and L_1_ smooth in some specimens). 8 + 8 ommatidia, G and H smaller; the eye H, seen at SEM, poorly developed externally (Fig 6B). Outer lobe of maxillary palp with four smooth chaetae in addition to the two basal chaetae (Fig 5J).

Body. Abd IV/III ratio 1.43–2.09 (mean 1.69, n = 13). Trochanteral organ with approximately 120 special chaetae with a segregate distribution of the longer chaetae (Fig 5B). Tibiotarsus apparently without sub-segmentation, although in some specimens, it was bent slightly beyond its midpoint; spine-like chaetae with appressed ciliation (apparently wide, smooth chaetae): 5–8 on leg 1; 10–15 on legs 2 and 3. Claw with one outer tooth at the level of a pair of stout lateral teeth and four inner teeth (two paired at 45–50%, a first unpaired at 75% and a minute unpaired at 85% of the internal edge total length). Empodial appendage lanceolate and concave with an outer tooth inserted at 30–35% of its total empodial length; empodial appendage/claw length ratio 0.67 (0.63–0.72, n = 4); smooth chaetae on tibiotarsus similar in length to empodial appendage (0.79–0.95, n = 4); tenent hair similar in length to empodial appendage (n = 4) (Fig 5H). Anterior face of ventral tube with many chaetae (>50), including 2+2 Mc on distal area; posterior face with >50 chaetae, including 1+1 Mc on latero-basal area; apical flaps with >50 smooth and ciliate chaetae.

Furcula. Males with manubrial organs with 3–4 small chaetae grouped similar to a brush. The smooth area of distal dens 2 times the mucronal length; basal spine on mucro reaching or slightly surpassing the basal tooth; teeth of similar size (Fig 5I).

Macrochaetotaxy (Figs 7B and 8B, Table 2). Head: H1 area with two An chaetae and seven more as mc; H2 area with four Mc and three mc; H3 area with S’_0_ present; H4 area with 2–3 Mc; the S series (from S_4i_ to the eye) with 10–13 chaetae, 1–2 as Mc; H5 area with two Mc.; H6 area with five Mc. Th II: T1 area with 7–9 Mc (n = 6); T2 area with 8 Mc. Th III with 2–3 Mc above pseudopore, and 13–22 chaetae in total (n = 3); lateral sensillae not identified. Abd I with three Mc around the pseudoporus (a_1_, a_2_ and m_2_) and 6–8 towards lateral; lateral sensillae not identified. Abd II with three Mc in A1 area (a_1_, a_2_ and another one) and four on A2 area (m_3_, m_3ei_, m_3e_ and m_3e2_); sensilla ‘as’ not identified; m_2_ and a_5_ as bothriotricha. Abd III with one Mc on A3 area (a_1_), three on A4 area (a_2_, a_2a_ and a_3_) and three on A5 area (m_3_, m_3e_ and m_3e2_; an additional, more external Mc was present in one specimen, male); sensilla ‘as’ above m_2_ (between m_3_ and a_3_); a_5_, m_2_ and a_5_ as bothriotricha, three Mc lateral to external bothriotricha. Abd IV: A6 area with two Mc (A_1_ and B_1_); A7 area with two Mc (B_2_ and C_1_); A8 area with four Mc (A_4_, A_4a_, Ae_4_ and B_4_); A9 area with two Mc (A_5_ and B_5_); and A10 area with only one Mc (A_6_).

Simplified Mc formula: 2(9)-4(7)-1-2(3)-2-5/7(9)-8/3-4/1-3-3/2-4-4-2-1.

S-chaetae formula: 2, 2/1, 5, 6, n, 14 (in Th II, in some specimens, acc.p6 duplicate; n = approximately 35, some of them four times the ‘normal’ S-chaetae). The ‘normal’ S-chaetae measured 24–26 micrometers, while the ‘long’ S-chaetae on Abd IV measured 95–100 micrometers. S-microchaetae formula 1, 0/1, 0, 1, 0, 0 (approximately 3.5 micrometers) (Fig 6E), respectively.

#### Biology

The study of the intestinal content of the specimens mounted on slides allowed for observing amorphous organic matter, although some pollen grains were also present in some specimens that likely belonged to *Pinus sylvestris*, which are common in the area.

#### Remarks

The coloration, which was an almost completely yellowish-white, of this species was similar to *O*. *flavescens*, which has two dorsolateral longitudinal lines from the head (behind the eyes) to the segment Abd VI in most specimens. It was possible to deduce part of the formula proposed in this work for *O*. *flavescens* using the information provided by Stach [28]:? -?-?-?-?/3-6/8-4/1-3-3/3-7(8)-3-2-0 (?, unknown), which makes it possible to differentiate it from *O*. *colluvialis* sp. nov. (Table 2) (other comparison among the different species of *Orchesella* cited in this paper is presented in Table 3). Other species recorded in Europe have some specimens from some populations with very little pigmentation, but they are always cited together with specimens with apparent pigmentation. In the case of *O*. *colluvialis* sp. nov., which was captured in hundreds of specimens in the sampling of this study, all specimens had pigmentation exclusively on the sides of the Abd VI (as shown in Fig 3D, there are very diffuse spots in other parts of the body, but that does not change the general color pattern of the species).

**Table 3:** Comparison of characters among the different species of *Orchesella* cited in this paper.

	*O*.* cincta*	*O*.* colluvialis* sp. nov.	*O*.* eolia*	*O*.* mesovoides* sp. nov.	*O*.* villosa*
***O*.* cincta***		13	10	12	7
***O*.* colluvialis* sp. nov.**	59%		13	10	7
***O*.* eolia***	46%	59%		12	10
***O*.* mesovoides* sp. nov.**	56%	46%	55%		9
***O*.* villosa***	31%	31%	46%	41%	

Up and right, number of characters (1 to 22 in Table 2) not shared between the compared species; left and down, percentage of dissimilarity between species.

**Fig 9:**
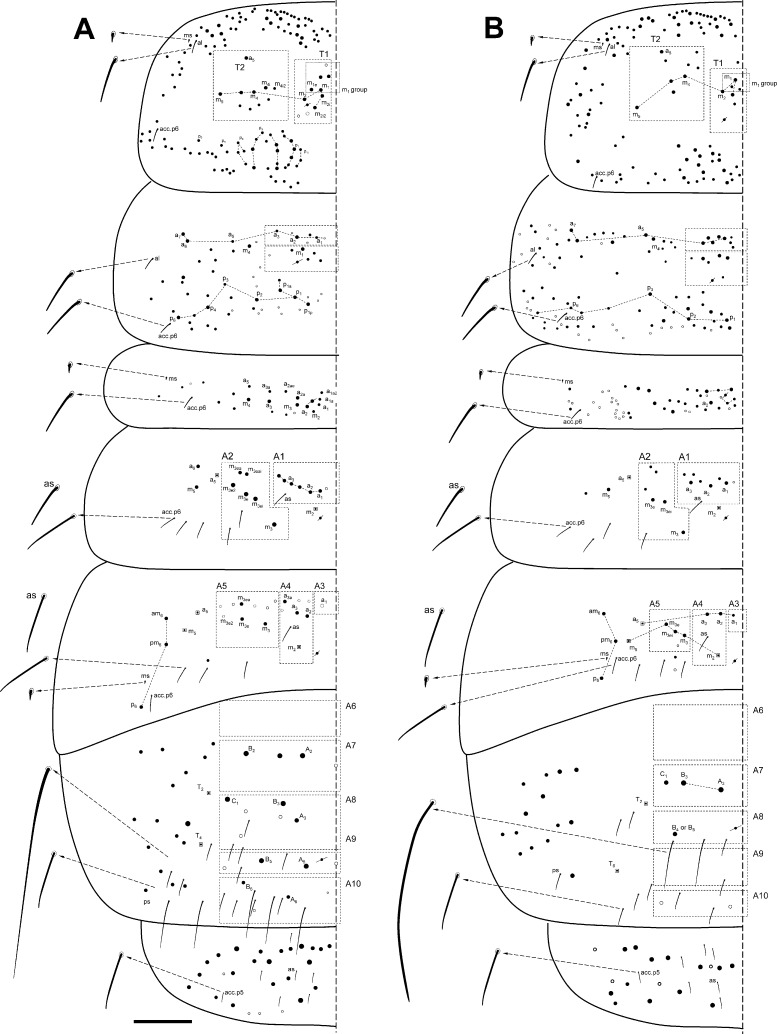
Dorsal macrochaetotaxy of Th II to Abd V. (A) *Orchesella villosa*. (B) *Orchesella cincta* (bar 0.2 mm; S-chaetae and S-microchaetae drawing in the lateral x10).

***Orchesella villosa*** Linnaeus, 1767

(Figs 3E, 4E, 5C, 5K and 5L, 7C and 9A)

Studied material.—Cizur Menor, Navarra, SPAIN, 22.IX.2016, *Pinus nigra* forest (on trunk), coordinates 42,789909,-1,676618, sample MZ-20160922a (slide 01, female; slide 02, juvenile), E. Baquero leg. Pamplona, Navarra, SPAIN, 19.I.2017, Campus of University of Navarra (garden), coordinates 42,804044,-1,663833, sample MZ-20170119a (slide 01, female; slide 02, female), E. Baquero leg.

#### Description

Body lengths of the specimens were 3.10–5.20 mm (mean 4.30 mm, n = 4) (Fig 3E).

Head. Ratio antennae/head = 3.05 (n = 2). Coloration is as described for the species [56]. First and second sub-segments of Ant I thick, some less than twice that of Ant II. Distal part of Ant III and Ant IV not annulated or verticillate, perhaps only a hint of annulation in the most distal part (Fig 4E) without an apical bulb. Labral papillae rounded, with pointed and curved tip, as a hook. Prelabral and labral chaetae all smooth (4/554). Lateral process (or differentiated chaeta) of outer labial palp (E) not reaching the middle of its papilla. Anterior labial row with four smooth and subequal chaetae; posterior labial row internal to chaeta E with 11–13 chaetae per side, all ciliated; chaetae E, L_1_ and L_2_ ciliated. 8+8 ommatidia, G and H smaller.

Body. Abd IV/III ratio 1.22 and 2.17 in the two specimens studied for this characteristic. Trochanteral organ with 95–100 special chaetae, with the longer situated only in a lateral direction (Fig 5C). Tibiotarsus without sub-segmentation; spiniform chaetae with appressed ciliation (apparently wide, smooth chaetae): 5–10 on leg 1; 10–15 on legs 2 and 3. Claw with one outer tooth, slightly more basal than the level of a pair of stout lateral teeth, and four inner teeth (two paired at 35–40%, a first unpaired at 65% and a last minute unpaired–absent in some specimens–at 85% of the internal edge total length). Empodial appendage lanceolated and concave with an outer tooth inserted at 30–35% of its total empodial length; empodial appendage/claw length ratio 0.60 (0.57–0.63, n = 4); smooth chaetae on tibiotarsus similar in length to empodial appendage (1.00–1.05, n = 4); tenent hair similar in length to empodial appendage (n = 4) (Fig 5K). Anterior face of ventral tube with many chaetae (>50), including 2+2 Mc on distal area; posterior face with >50 chaetae, including 2+2 Mc on latero-basal area; apical flaps with >50 smooth and ciliate chaetae.

Furcula. Males with manubrial organ with some chaetae grouped similar to a brush. The smooth area of distal dens was 1–2 times the mucronal length; basal spine on mucro reaching or slightly surpassing the basal tooth; teeth of similar size (Fig 5L).

Macrochaetotaxy (Figs 7C and 9A, Table 2). Head: H1 area with four An chaetae, some more as mc; H2 area with three Mc and 2–3 mc; H3 area with S’_0_ present; H4 area with two Mc; the S series (from S4i to the eye) with 10–12 chaetae, 5–6 as Mc; H5 area with three Mc; H6 area with five Mc. Th II: T1 area with seven Mc (n = 4), and 1–2 additional mc; T2 area with 2–3 Mc and 2–5 additional mc. Th III with 2–3 Mc above pseudoporus, and 10–11 additional mc (n = 2); lateral sensillae not identified. Abd I with three Mc around the pseudoporus (a_2_, a_2a_ and m_3_), and 2–3 towards lateral direction; lateral sensillae not identified. Abd II with six Mc in A1 area (a_1_, a_2_ and probably a_3_ presents) and five on area A2 (m_3_, m_3ei_, m_3e_, m_3e2_ and m_3eai_); sensilla ‘as’ not identified; m_2_ and a_5_ as bothriotricha. Abd III with or without Mc on A3 area (a_1_), three on A4 area (a_2_, a_3_ and a_3a_) and three on A5 area (m_3_, m_3e_ and m_3ea_); sensilla ‘as’ above m_2_ (slightly behind the line joining m_3_ and a_3_); a_5_, m_2_ and a_5_ as bothriotricha, and three Mc lateral to external bothriotricha. Abd IV: A6 area without Mc; area A7 with (A_2_ and B_2_); A8 area with three Mc (A_3_, B_3_, and C_1_); areas A9 and A10 without Mc, only with some mc.

Simplified Mc formula: 4(8)-3(5)-1-2-3-5/7-5/6-5(6)/0(1)-3-3/0-3-3-2-2.

S-chaetae formula: 2, 2/1, 6, 5, n, 7 (n = 17, five of them four times the ‘normal’ S-chaetae). Th II and Abd III with one S-microchaetae (approximately 3.5 micrometers), respectively. The ‘normal’ S-chaetae measured 24–26 micrometers, while the ‘long’ S-chaetae on Abd IV measured 95–100 micrometers. S-microchaetae formula 1, 0/1, 0, 1, 0, 0 (approximately 3.5 micrometers), respectively (Fig 9A).

#### Remarks

This species has a wide distribution in the Northern Hemisphere. The macro-chaetotaxy contributed to this paper.

***Orchesella cincta*** Linnaeus, 1758

(Figs 3F, 4F and 4G, 5D, 5M and 5N, 7D and 9B)

Studied material.—Agüero-Marina de Cudeyo, Cantabria, SPAIN, 12.10.1991, Solaheza cave, coordinates (WGS84) 30T VP 441490 4807564 z = 25 (43.41868123,-3.72276624, 25 m), sample A-88 01 (female), C. González-Luque leg. Prellezo–Val de San Vicente, Cantabria, SPAIN, 25.VII.1992, Cueva del Rojo (Red’s cave), coordinates 30T UP 382743 4804399 z = 70 (43.38330352,-4.44761079, 70 m), sample A-108 (slide 01, female; slide 02, female; slide 03, male), C. González-Luque leg. Escobedo-Camargo, Cantabria, SPAIN, 20.VII.1991, Covachos I de Peñajorao, coordinates 30T VP 425723 4804264 z = 52 (43.38757361,-3.91706365, 52 m), sample A-68 BIS 01 (female), C. González-Luque leg. Lizoáin, Navarra, SPAIN, 25.IX.2014, Erro River (Aizpuru-El Puente), coordinates 42.821200, -1.457666, sample MZ-20140925f (slide 01, female; slide 02 female), E. Baquero leg.

#### Description

The body lengths of the specimens studied were 3.65–4.40 mm (mean 4.00 mm, n = 7) (Fig 3F).

Head. Ratio antennae/head = 3.18 (n = 3). Coloration is as described for the species [56]. First and second sub-segments of Ant I wider than Ant II. Distal part of Ant III and Ant IV not annulated or verticillate, perhaps only a hint of annulation in the most distal part (Fig 4F and 4G), without an apical bulb. Labral papillae rounded, with pointed and curved tip, as a hook. Prelabral and labral chaetae all smooth (4/554). Lateral process (or differentiated chaeta) of outer labial palp (E) not reaching the middle of its papilla. Anterior labial row with four smooth and subequal chaetae; posterior labial row internal to chaeta E with 7–9 chaetae per side, all ciliated; chaetae E and L_1_ ciliated, L_2_ smooth. 8 + 8 ommatidia, G and H smaller.

Body. Abd IV/III ratio 1.30–2.11 (n = 7). Trochanteral organ with 80–90 special chaetae (Fig 5D). Tibiotarsus without sub-segmentation, although in some specimens, it was bent slightly beyond its midpoint; spiniform chaetae with appressed ciliation (apparently wide, smooth chaetae): 5–10 on leg 1; 10–12 on legs 2 and 3. Claw with one outer tooth, slightly more basal than the level of a pair of stout lateral teeth, and four inner teeth (two paired at 40%, a first unpaired at 70% and a last unpaired, with a significant development at 90% of the internal edge total length). Empodial appendage lanceolated and concave, with an outer tooth inserted at 50% of its total empodial length; empodial appendage/claw length ratio 0.67 (0.64–0.70, n = 4); smooth chaetae on tibiotarsus similar in length to empodial appendage (0.97–1.03, n = 4); tenent hair slightly longer (1,27 times) in length than empodial appendage (n = 1) (Fig 5M). Anterior face of ventral tube with many chaetae (>50), including 2+2 Mc on distal area; posterior face with >50 chaetae, including 1+1 Mc on middle-posterolateral area, and 2+2 ms on basal area; apical flaps with >50 smooth and ciliate chaetae, some of them bigger than other.

Furcula. Males with manubrial organs with four chaetae grouped similar to a brush. The smooth area of distal dens 3–4 times the mucronal length (although in one of the studied specimens, it was only nearly 2); basal spine on mucro reaching or slightly surpassing the basal tooth; distal tooth bigger than basal (Fig 5N).

Macrochaetotaxy (Figs 7D and 9B, Table 2). Head: H1 area with five An Mc; H2 area with three Mc; area H3 without S’_0_; H4 area with two Mc; the S series (from S_4i_ to the eye) with 10 Mc and two mc; H5 area with one Mc (Ps_2_); H6 area with two Mc. Th II: area T1 with three Mc (n = 1), and 2–3 additional mc; T2 area with five Mc and four additional mc (n = 1). Th III with seven Mc above pseudoporus, and 6–7 additional mc (n = 1); lateral sensillae not identified. Abd I with three Mc around the pseudoporus (a_2_ and another two), and 3–4 towards the lateral direction (the difference in size between the insertions of Mc and mc was relative); lateral sensillae not identified. Abd II with three Mc in A1 area (a_1_, a_2_ and a_3_), and three on A2 area (m_3_, m_3ei_ and m_3e_); sensilla ‘as’ not identified; m_2_ and a_5_ as bothriotricha. Abd III with Mc on A3 area (a_1_)—almost a mc—two on A4 area (a_2_ and a_3_) and three on A5 area (m_3_, m_3e_ and m_3ei_); sensilla ‘as’ not identified; a_5_, m_2_ and a_5_ as bothriotricha, and three Mc lateral to external bothriotricha. Abd IV: area A6 without Mc; A7 area with three Mc (A_3_, B_3_ and C_1_); A8 area with one Mc (B_4_ or B_5_); A9 area without Mc; and ara A10 with only two mc.

Simplified Mc formula: 5-3-0-2-1b-2/7-7/4-3/1-2-3/0-3-1-0-0(2*) (m_5_ in Th II is present) (*meaning mesochaetae).

S-chaetae. Formula: 2, 2/1, 5, 5, n, 7 (n = 12, two of them four times the ‘normal’ S-chaetae; the pigmentation of the specimens observed) (the pigmentation of the specimens makes it very difficult to see the S-chaetae in most specimens). The ‘normal’ S-chaetae measured 24–26 micrometers, while the ‘long’ S-chaetae on Abd IV measured 95–100 micrometers. S-microchaetae formula 1, 0/1, 0, 1, 0, 0 (approximately 3.5 micrometers), respectively (Fig 9B).

#### Remarks

The specimens studied were identified by color. In this study, the information regarding the macro-chaetotaxy of this species is presented for the first time, which will allow for comparisons when studying the specimens identified by other researchers either in the past or in the future. A wide distribution may be attributed to the species based only on coloration changes when the macro-chaetotaxy is used.

**Fig 10:**
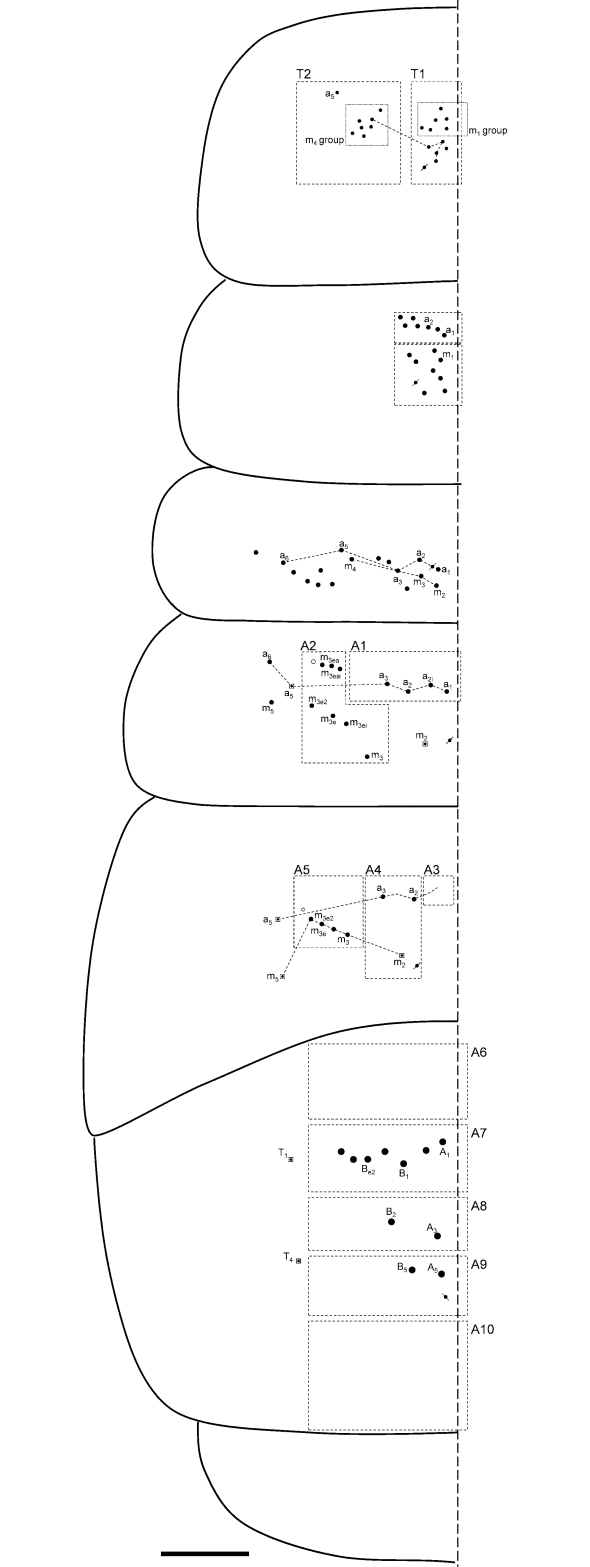
Dorsal macrochaetotaxy of Th II to Abd V of *Orchesella eolia* (bar 0.2 mm).

***Orchesella eolia*** Altner, 1961

(Figs 3C, 7F and 10)

Studied material.—Stromboli Island, Italy, five specimens (paratypes) in ethyl alcohol from MHNG, code sample 7412 STR and 7412a STR. In two specimens, the color pattern was visible (Fig 3C). The specimen with more damaged pigmentation was cleared (KOH) and mounted in Hoyer’s medium.

Simplified Mc formula: 5-4(3*)-0-2-1-3/11-8/4-7/0-2-4/0-7-2-2-0 (Figs 7F and 10, Table 2).

### Ecology

**Fig 11:**
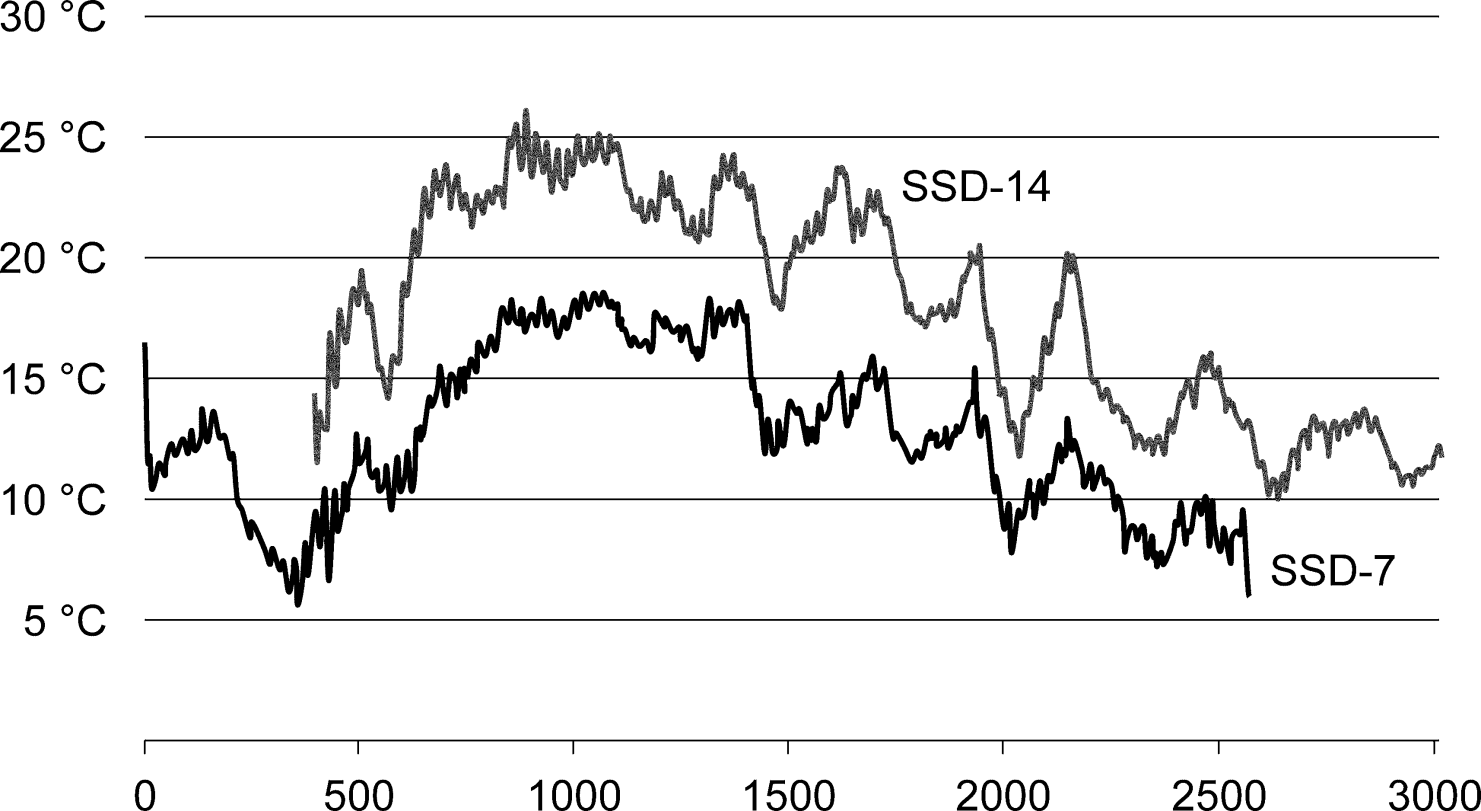
MSS temperature. Temperature records of two of the SSDs, one (SSD-14) was the highest in Peñalara (Majada Hambrienta) (1994 m a. s. l.), and the other (SSD-7) was the lowest in El Purgatorio (1406 m a. s. l.). x-axis: number of measures from June to September; y-axis: temperature in Celsius degrees.

**Fig 12:**
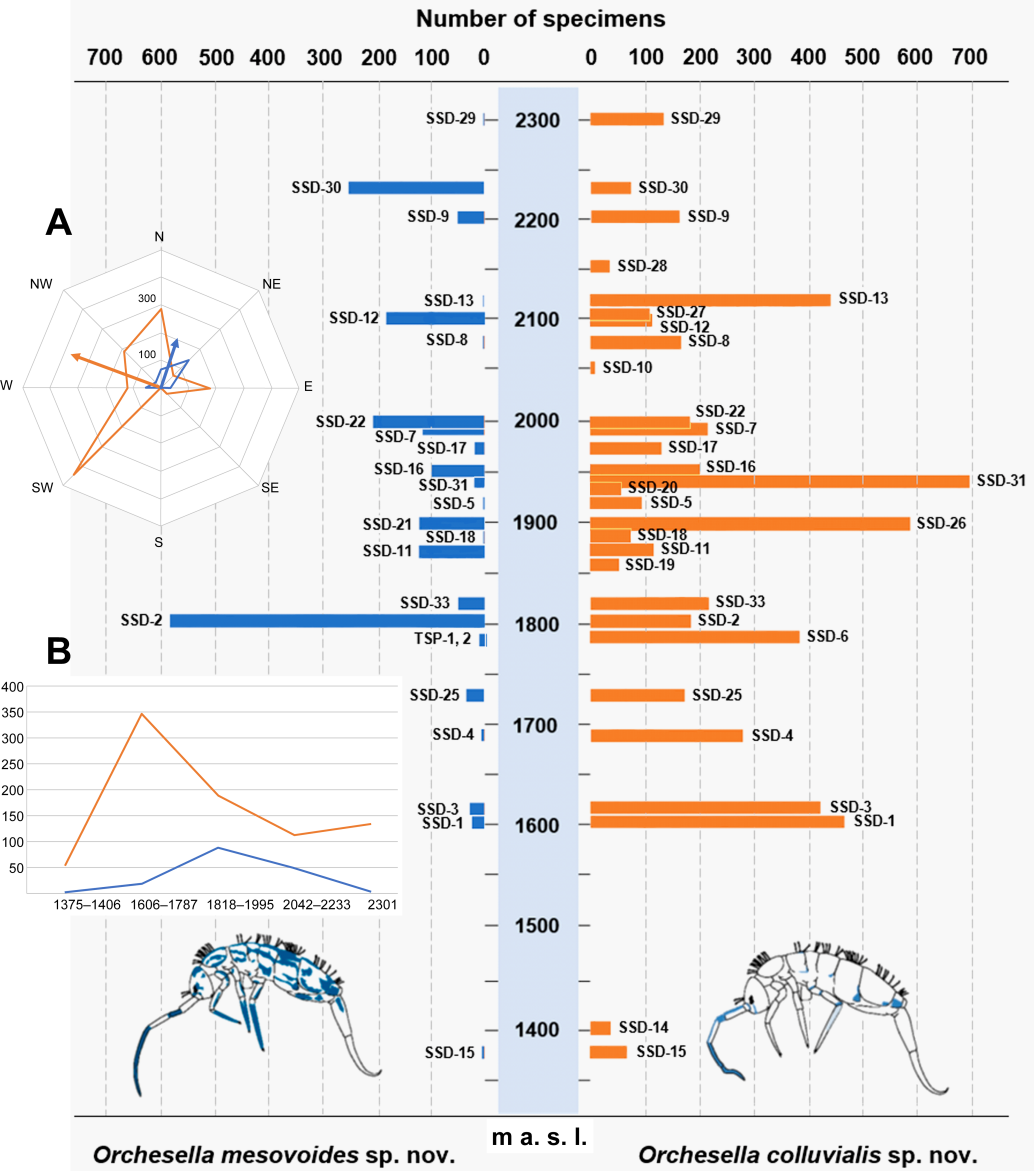
Number of specimens of the two *Orchesella* species described in this paper ordered by altitude. (A) Geographical orientation of the vectors of abundance of the two species. (B) Abundance of the species in relation to altitude (orange: *O*. *colluvialis* sp. nov.; blue: *O*. *mesovoides* sp. nov.).

The study allowed for obtaining data regarding the microclimatic conditions of the sampled habitat, mainly of temperature, during the sampling period from June to September of 2016. It was observed that the highest monthly average temperatures were recorded in July, with 17° C in the highest altitudes and 23° C in the lowest altitudes, which decreased below 15° C in late August. The temperatures in the MSS never exceeded 26° C and did not decrease below 5° C during the cited sampling period. In addition, a temperature range between 5 and 18° C was observed in the traps located in the surroundings of 2000 m. a. s. l., and in the surroundings of 1500 m. a. s. l., the temperature range was between 10 and 25° C (Fig 11). The presence of the two species along the Sierra de Guadarrama was fairly uniform (Table 4); however, it was observed that *O*. *colluvialis* sp. nov. seemed to prefer a range of higher altitudes than *O*. *mesovoides* sp. nov. Considering the average number of animals per trap (to weigh the number of traps placed by height), it was observed that the maximum abundance for the species *O*. *colluvialis* sp. nov. was between 1600 and 1800 m. a. s. l., while *O*. *mesovoides* sp. nov., which was always found in lower abundances, had a maximum abundance between 1800 and 2000 m. a. s. l., indicating a preference for higher altitudes (Fig 12 and inset A). Considering the average number of animals per trap (to weigh the quantity of traps per orientation), it was observed that the vector sum of the abundances for the species *O*. *colluvialis* sp. nov. was located in the W-NW orientation, whereas for the species *O*. *mesovoides* sp. nov., it was N-NE (Fig 12, inset B).

**Table 4:** Captures for the species *O*. *mesovoides* sp. nov. and *O*. *colluvialis* sp. nov. in all the traps.

Siete Picos - La Mujer Muerta	Sample	SSD-1 (0,5)	SSD-1	SSD-2 (0,5)	SSD-2	SSD-3 (0,5)	SSD-3	SSD-4 (0,5)	SSD-4	SSD-11							
	altitude	1606	1606	1818	1818	1622	1622	1685	1685	1876							
	***O*.* mesovoides* sp. nov.**	6	20	114	468	0	29	0	8	123							
	***O*.* colluvialis* sp. nov.**	260	207	17	170	425	0	0	279	117							
Puerto de los Cotos - Puerto de Navacerrada	Sample	SSD-5	SSD-6														
	altitude	1923	1787														
	***O*.* mesovoides* sp. nov.**	2	0														
	***O*.* colluvialis* sp. nov.**	96	389														
Montes Carpetanos	Sample	SSD-7	SSD-8	SSD-9	SSD-10	SSD-16	SSD-17	SSD-18	SSD-19	SSD-20	SSD-21	SSD-22	SSD-23	SSD-24	SSD-25	TSP-1	TSP-2
	altitude	1994	2071	2208	2049	1956	1976	1885	1866	1937	1891	1995	2144	2042	1731	1780	1780
	***O*.* mesovoides* sp. nov.**	116	4	50	0	100	20	3	0	0	120	206	0	0	35	12	1
	***O*.* colluvialis* sp. nov.**	216	168	163	10	201	132	75	55	57	0	184	0	0	173	0	0
Cuerda Larga and Associated Mountainous complex	Sample	SSD-12	SSD-13	SSD-14	SSD-15	SSD-26	SSD-27	SSD-28	SSD-29	SSD-30	SSD-31	SSD-32	SSD-33				
	altitude	2102	2113	1406	1375	1890	2101	2156	2301	2233	1946	1948	1819				
	***O*.* mesovoides* sp. nov.**	184	2	0	6	1	0	0	4	252	20	0	50				
	***O*.* colluvialis* sp. nov.**	115	443	40	68	591	112	38	135	73	700	0	220				
		Total															
Guadarrama (all traps)	***O*.* mesovoides* sp. nov.**	1956															
	***O*.* colluvialis* sp. nov.**	5929															

The presence of pollen granules in the species *O*. *colluvialis* sp. nov. may be related to its more abundant presence in lower areas of the mountain range where the tree vegetation, *Pinus sylvestris*, is more abundant.

The two new species described have similarities with respect to their distribution and abundance at different places, but it can be argued that the species *O*. *colluvialis* sp. nov. seems to be more adapted to the underground environment, which could be based on several factors: its coloration with little pigment, a greater reduction of the eyes (the G and H eyes was far more reduced in the optical microscope, which was confirmed when observed at SEM) (Fig 6B) and more S-chaetae. *O*. *mesovoides* sp. nov. had been referenced from Sierra de Guadarrama (erroneously identified as *O*. *eolia*), and given the lack of previous samplings in the subterranean environment, these references suggest that this species has less dependence on the subterranean habitat. Nevertheless, it has never been recorded at the surface.

The abundance by trap varied between a few specimens and at a maximum of 700 (in this case, the species *O*. *colluvialis* sp. nov.). For traps with two-level catches, no difference was observed for the two species described. In total, 1956 specimens of *O*. *mesovoides* sp. nov. and 5929 specimens of *O*. *colluvialis* sp. nov. were caught. The data confirmed that the species are abundant in the massif. They have not been captured previously, with the exception of the capture of the specimens identified as *O*. *eolia* by Selga in Collado Alto and Valsaín, which demonstrates their absence on the surface in this area. Based on the findings it can be affirmed that the *Orchesella* communities of the Colluvial MSS of Sierra de Guadarrama have their own species. Interestingly, other *Orchesella* species appeared to inhabit the Colluvial MSS in other regions of Europe [16, 17, 22] and even in the Iberian Peninsula [12, 14]. Thus, future works should reveal a significant hidden diversity of species of the genus as well as different species assemblages in the MSSs of different regions.

## Discussion

Six species of *Orchesella* have been referenced for the Iberian Peninsula thus far; however, one (*O*. *eolia*) was considered a misidentification after study completion. Only four have been cited in the area of Sierra de Guadarrama and surroundings by conducting surface fauna sampling: *O*. *cincta* (Linnaeus, 1758) (San Lorenzo de El Escorial, Madrid [55]), *O*. *flavescens* (Sierra de Guadarrama, Cercedilla, Madrid [55]; Valsaín, Sierra de Guadarrama, Madrid [55]), *O*. *quinquefasciata* (Navacerrada, Sierra de Guadarrama, Madrid [55]; Cercedilla, Sierra de Guadarrama, Madrid [55]; San Lorenzo de El Escorial, Madrid [55]; Navacepeda de Tormes, oak forest, Sierra de Gredos[57]; Sierra de Gredos, pine forest litter [58]; Montejo de la Sierra, beech forest, Mazizo de Ayllón [59]), *O*. *villosa* (Los Cotos and Navacerrada, Sierra de Guadarrama, Madrid [55]).

In addition to the work of the researchers cited above and to Bonet and Acon [60, 61], who collected Collembola from 1929 until recently, and with the exception of two specimens identified as *O*. *eolia* found in a gallery of a mine, there have not been any additional registers of the *Orchesella* species described in this paper. Nevertheless, they have been proven to be abundant in the colluvial MSS.

It was possible to compare the two new species described in this paper with the species *O*. *villosa*, *O*. *cincta* and *O*. *eolia* (Table 3). The macrochaetotaxy of *O*. *flavescens sensu* Stach [28] has been studied for comparisons with *O*. *colluvialis*. It was not possible to study the species *O*. *quinquefasciata*. While comparing the macrochetotaxy and other morphological characters, it was found that with these characteristics and without color, these species can be differentiated. This is of enormous importance because until now, the identification of the genus *Orchesella* was based almost only on the coloration. Moreover, the sensilar chaetotaxy (S-chaetae and S-microchaetae) was examined in detail, since it has been used for other genera [62], although it is difficult to observe in species with large specimens.
